# The Ancestral N-Terminal Domain of Big Defensins Drives Bacterially Triggered Assembly into Antimicrobial Nanonets

**DOI:** 10.1128/mBio.01821-19

**Published:** 2019-10-22

**Authors:** Karine Loth, Agnès Vergnes, Cairé Barreto, Sébastien N. Voisin, Hervé Meudal, Jennifer Da Silva, Albert Bressan, Nawal Belmadi, Evelyne Bachère, Vincent Aucagne, Chantal Cazevielle, Hélène Marchandin, Rafael Diego Rosa, Philippe Bulet, Lhousseine Touqui, Agnès F. Delmas, Delphine Destoumieux-Garzón

**Affiliations:** a Centre de Biophysique Moléculaire UPR4301 CNRS, Orléans, France; b UFR CoST, Université d’Orléans, Orléans, France; c IHPE, Université de Montpellier, CNRS, Ifremer, Université de Perpignan Via Domitia, Montpellier, France; d Laboratory of Immunology Applied to Aquaculture, Department of Cell Biology, Embryology and Genetics, Federal University of Santa Catarina, Florianópolis, Santa Catarina, Brazil; e Plateforme BioPark d’Archamps, Archamps Technopole, Archamps, France; f Equipe mixte Institut Pasteur/Paris V Mucoviscidose et Bronchopathies Chroniques, Institut Pasteur, Paris, France; g COMET, Plateau de microscopie électronique, Plateforme Montpellier RIO Imaging, Montpellier, France; h HydroSciences Montpellier, Département de Microbiologie, CHU Nîmes, CNRS, IRD, Université de Montpellier, Nîmes, France; i Institute for Advanced Biosciences, CR Université Grenoble Alpes, CNRS UMR5309, La Tronche, France; University of Texas at Austin; University of Georgia

**Keywords:** MRSA, antimicrobial peptides, antimicrobial resistance, defensins, fibrils, innate immunity, mechanisms of action, nuclear magnetic resonance

## Abstract

Big defensins, ancestors of β-defensins, are composed of a β-defensin-like C-terminal domain and a globular hydrophobic ancestral N-terminal domain. This unique structure is found in a limited number of phylogenetically distant species, including mollusks, ancestral chelicerates, and early-branching cephalochordates, mostly living in marine environments. One puzzling evolutionary issue concerns the advantage for these species of having maintained a hydrophobic domain lost during evolution toward β-defensins. Using native ligation chemistry, we produced the oyster Crassostrea gigas BigDef1 (*Cg-*BigDef1) and its separate domains. *Cg-*BigDef1 showed salt-stable and broad-range bactericidal activity, including against multidrug-resistant human clinical isolates of Staphylococcus aureus. We found that the ancestral N-terminal domain confers salt-stable antimicrobial activity to the β-defensin-like domain, which is otherwise inactive. Moreover, upon contact with bacteria, the N-terminal domain drives *Cg-*BigDef1 assembly into nanonets that entrap and kill bacteria. We speculate that the hydrophobic N-terminal domain of big defensins has been retained in marine phyla to confer salt-stable interactions with bacterial membranes in environments where electrostatic interactions are impaired. Those remarkable properties open the way to future drug developments when physiological salt concentrations inhibit the antimicrobial activity of vertebrate β-defensins.

## Abstract (precis)

β-Defensins are host defense peptides controlling infections in species ranging from humans to invertebrates. However, the antimicrobial activity of most human β-defensins is impaired at physiological salt concentrations. We explored the properties of big defensins, the β-defensin ancestors, which have been conserved in a number of marine organisms, mainly mollusks. By focusing on a big defensin from oyster (*Cg-*BigDef1), we showed that the N-terminal domain lost during evolution toward β-defensins confers bactericidal activity to *Cg-*BigDef1, even at high salt concentrations. *Cg-*BigDef1 killed multidrug-resistant human clinical isolates of Staphylococcus aureus. Moreover, the ancestral N-terminal domain drove the assembly of the big defensin into nanonets in which bacteria are entrapped and killed. This discovery may explain why the ancestral N-terminal domain has been maintained in diverse marine phyla and creates a new path of discovery to design β-defensin derivatives active at physiological and high salt concentrations.

## INTRODUCTION

β-Defensins are essential components of innate immunity broadly found in vertebrates and invertebrates (1). Through their multiple functions (e.g., as antimicrobial peptides [AMPs], proinflammatory mediators of the immune response), these cationic host defense peptides (HDPs) contribute to protection against infections at almost all human epithelial surfaces (2, 3). Some human β-defensins were shown to compromise bacterial membrane integrity (4). However, like many human (5–7) and avian (8) β-defensins, direct antimicrobial activity at physiological salt concentrations is significantly impaired. This suggests that electrostatic interactions initiating interactions between these cationic peptides and the negatively charged membranes of bacteria (9) are altered by salts.

Big defensins are a group of AMPs related to β-defensins, initially isolated from the hemocytes of a marine chelicerate, the horseshoe crab (Tachypleus tridentatus) (*Tt-*BigDef) (10). They contain a N-terminal hydrophobic domain and a C-terminal β-defensin-like domain (11). The two domains are encoded by separate exons (12, 13). On the basis of their gene structure and amino acid sequence and three-dimensional (3D) structure similarities, it was proposed that vertebrate β-defensins originated from an ancestral big defensin via intronization of exonic sequences or exon shuffling, thereby losing the ancestral N-terminal domain (1, 13). Big defensins have been predominantly described in marine organisms, mainly mollusks (12, 14), and, to a much lower extent, in ancestral chelicerates (horseshoe crabs) (10) and early-branching chordates (amphioxus) (15). The evolutionary advantage for those phylogenetically distant species of having conserved the N-terminal hydrophobic domain represents one puzzling unsolved issue.

Although they were isolated more than 20 years ago (10), technical limitations impeding the production of sufficient quantities of big defensins have precluded investigations on their structure and antimicrobial activities and of the roles of their respective domains. Sufficient amounts of native *Tt-*BigDef were obtained from horseshoe crab hemocytes to determine its 3D structure (Protein Data Bank [PDB] identifier: 2RNG), which remains the only one solved to date. *Tt-*BigDef is tightly packed in solution: the hydrophobic N-terminal domain adopts a β1-α1-α2-β2 fold, whereas the cationic C-terminal domain shows the cysteine pairing expected for β-defensins (11). To date, data on big defensin antimicrobial activities remain scarce (10, 14, 15).

Achievements that opened the way to structure and activity studies in big defensins included developments in peptide chemistry related to native chemical ligation (NCL) strategies (16, 17). Using this methodology, we synthetized *Cg-*BigDef1, one of the best-characterized big defensins in terms of gene structure, expression, and role in immunity (12, 18–20). We obtained several dozen milligrams, solved its solution structure, and investigated its interactions with bacteria. By using a combination of antimicrobial assays, immune detection, scanning electron microscopy, and mass spectrometry analyses, we determined that *Cg-*BigDef1 is a highly salt-stable AMP that entraps and kills bacteria in nanonet structures. Moreover, we found that *Cg-*BigDef1 is (i) active against clinical strains of Staphylococcus aureus multiresistant to antibiotics, which represent a major concern for human health, and (ii) devoid of cytotoxicity toward mammalian cells. Our findings pave the way for future drug developments inspired by the evolution-based molecular design of big defensins.

## RESULTS

### Big defensins are mainly found in marine organisms.

**FIG 1:**
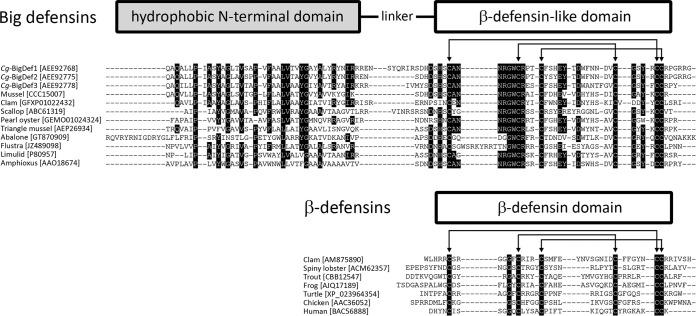
Amino acid sequence alignments of big defensins and β-defensins. Conserved residues are highlighted in black. Arrows indicate the six cysteine residues that follow the canonical cysteine spacing of β-defensins and big defensins. The schematic representation (not to scale) shown at the top of the alignments indicates the structural domain organization of mature big defensins and β-defensins. Cysteine pairing is indicated by black lines based on previously reported data (10, 53) and our NMR data (this study).

We performed an exhaustive search for sequences containing a β-defensin domain in publicly available genomic and transcriptomic databases. Sequences of β-defensins were found in diverse groups of vertebrates (from fish to mammals) and invertebrates (mollusks and crustaceans), whereas big defensins were present in a limited number of species belonging to Lophotrochozoa, Arthropoda, and Cephalochordata (Fig. 1). Remarkably, of the 78 obtained distinct big defensins, only one sequence (GenBank accession no. AEP26934) was found in a nonmarine species, the sequence corresponded to the freshwater mussel Hyriopsis cumingii (21). Mollusks (Lophotrochozoa) represented the superphylum containing the highest diversity of big defensins by far (see Fig. S1 in the supplemental material). Multiple-sequence alignments revealed a canonical conserved motif but differently spaced cysteines for big defensins [Cys-Xaa_(4–14)_-Cys-Xaa_(3)_-Cys-Xaa_(13–14)_-Cys-Xaa_(4–7)_-Cys-Cys] and β-defensins [Cys-Xaa_(4–6)_-Cys-Xaa_(3–4)_-Cys-Xaa_(7–12)_-Cys-Xaa_(5–7)_-Cys-Cys] (Fig. 1). The two defensin families also differed by the presence of a hydrophobic N-terminal domain (20 to 64 residues) in big defensins only (Fig. 1). This domain, which contains some highly conserved amino acids (Fig. 1), does not show any homology with sequences present in public databases outside big defensins.

10.1128/mBio.01821-19.2 FIG S1 Multiple amino acid sequence alignments of big defensins (Lophotrochozoa, Arthropoda, and Cephalochordata) with β-defensins from both vertebrates (from fish to mammals) and invertebrates (mollusks and crustaceans). Conserved residues and cysteines are highlighted in gray and black, respectively. Download FIG S1, DOCX file, 1.1 MB. Copyright © 2019 Loth et al. 2019 Loth et al. This content is distributed under the terms of the Creative Commons Attribution 4.0 International license.

### Native chemical ligation-based chemical synthesis gives access to the exploration of multiple-domain *Cg-*BigDef1 structure and activity.

**FIG 2:**
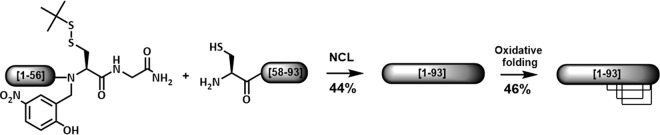
Synthetic scheme of *Cg-*BigDef1. The N-terminal cysteine-containing peptide *Cg*-BigDef1[58-93] (CANNRGWCRPTCFSHEYTDWFNNDVCGSYRCCRPGRR) and the *Cg*-BigDef1[1–56] peptide (ZAQALLPIASYAGLTVSAPVFAALVTVYGAYALYRYNIRRRENSYQRIRSDHDSHS [“Z” being pyroglutamic acid]) equipped with our thioesterification device [(Hnb)Cys(S*t*Bu)-Gly-NH_2_] reacted under standard NCL conditions (22). After purification, the reduced form of *Cg*-BigDef1[1–93] was engaged in oxidative folding under thermodynamical control conditions (see Fig. S2). Percentages represent yields after HPLC purification.

To explore the role of the ancestral N-terminal domain in big defensin structure and activity, we focused on the mollusk big defensin *Cg-*BigDef1 (GenBank accession no. AEE92768). We first synthesized the entirety of *Cg-*BigDef1. *Cg*-BigDef1[1–93] corresponds to mature *Cg-*BigDef1 with a pyroglutamic acid (Pca) at the N terminus, an amidated C terminus, and six cysteines involved in three disulfide bridges (12) (Table 1). Total synthesis of *Cg-*BigDef1 (Fig. 2) was achieved through NCL (16) of *Cg*-BigDef1[57–93] with a *Cg*-BigDef1[1–56] cryptothioester (22) and subsequent oxidative folding (Fig. S2d). The resulting peptide was characterized by liquid chromatography-mass spectrometry (LC-MS) (Fig. S2e) and nuclear magnetic resonance (NMR) spectroscopy (see below). *Cg*-BigDef1[1–42] and *Cg*-BigDef1[44–93], which correspond to the N-terminal and C-terminal domains of *Cg-*BigDef1, respectively (Table 1), were synthesized by solid-phase peptide synthesis according to the position of *Cg-bigdef1* exons (12) (Fig. S2a to c).

**TABLE 1:** Sequence of *Cg-*BigDef1 and its two separated domains

Name	Sequence^*a*^
*Cg*-BigDef1[1–93]	ZAQALLPIASYAGLTVSAPVFAALVTVYGAYALYRYNIRRRENSYQRIRSDHDSHSCANNRGWCRPTCFSHEYTDWFNNDVCGSYRCCRPGRR-NH_2_
*Cg*-BigDef1[1–42]	ZAQALLPIASYAGLTVSAPVFAALVTVYGAYALYRYNIRRRE-NH_2_
*Cg*-BigDef1[44–93]	SYQRIRSDHDSHSCANNRGWCRPTCFSHEYTDWFNNDVCGSYRCCRPGRR-NH_2_

a Proteogenic amino acids are abbreviated using the one-letter code; Z is the pyroglutamic acid or pyrrolidinocarboxilic acid (also abbreviated “Pca” with the three-letter code); the C terminus is amidated. The underlined cysteinyl residues (C) are involved in disulfide bonds.

10.1128/mBio.01821-19.3 FIG S2 Peptide synthesis. (a) HPLC trace of purified *Cg*-BigDef1[1–42]. A Chromolith RP C_18_ column was used as follows: flow rate, 3 ml/min; solvent A = 0.1% TFA–water; solvent B = 0.1% TFA–CH_3_CN; gradient of solvent B in solvent A, 5% to 50% over 5 min. (b) Analytical monitoring of the *Cg*-BigDef1[44–93] oxidative folding by HPLC. A Chromolith RP C_18_ column was used as follows: flow rate, 3 ml/min; solvent A = 0.1% TFA–water; solvent B = 0.1% TFA–CH_3_CN; gradient of solvent B in solvent A, 15% to 40% over 5 min. (c) HPLC trace of purified *Cg*-BigDef1[44–93]. A Chromolith RP C_18_ column was used as follows: flow rate, 3 ml/min; solvent A = 0.1% TFA–water; solvent B = 0.1% TFA–CH_3_CN; gradient of solvent B in solvent A, 5% to 50% over 5 min. (d) Analytical monitoring of *Cg*-BigDef1[1–93] oxidative folding by HPLC. For panel A, a Jupiter RP C4 column was used as follows: flow rate, 1 ml/min; solvent A = 0.1% TFA–water; solvent B = 0.1% TFA–CH_3_CN; gradient of solvent B in solvent A, 30% to 80% over 30 min at 70°C. Blue trace, HPLC-purified reduced form of *Cg*-BigDef1[1–93] before oxidative folding; red trace, after 16 h reaction; green trace, after 40 h reaction. For panel B, ESI-HR-MS of the reduced form of *Cg*-BigDef1[1–93] found 10,691.0918 (calculated for C_467_ H_702_ N_146_ O_134_ S_6_ 10,691.0930 Da monoisotopic mass]). (e) HPLC and MS characterization of purified oxidized form of *Cg*-BigDef1[1–93]. Panel A, HPLC trace of purified *Cg*-BigDef1[1–93]. A Chromolith RP C_18_ column was used as follows: flow rate, 3 ml/min; solvent A = 0.1% TFA–water; solvent B = 0.1% TFA–CH_3_CN; gradient of solvent B in solvent A, 30% to 80% over 6 min. Panel B, ESI-HR-MS of the oxidized form of *Cg*-BigDef1[1–93]: found 10,685.0402 (calculated for C_467_ H_696_ N_146_ O_134_ S_6_ 10,685.0460 Da [monoisotopic mass]). Download FIG S2, DOCX file, 0.5 MB. Copyright © 2019 Loth et al. 2019 Loth et al. This content is distributed under the terms of the Creative Commons Attribution 4.0 International license.

### *Cg-*BigDef1 adopts a highly compact hydrophobic fold in solution.

**FIG 3:**
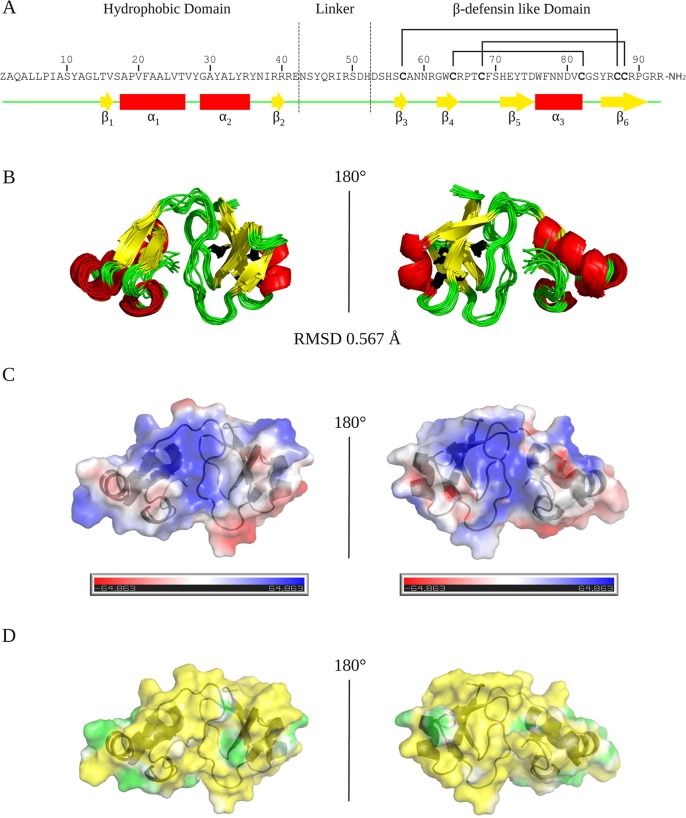
Solution structure of *Cg-*BigDef1 (PDB entry 6QBL). The global fold and surface potentials of *Cg*-BigDef1[1–93] are displayed. (A) *Cg*-BigDef1[1–93] sequence. “Z” stands for pyroglutamic acid (Pca) and NH_2_ for the C-terminal amidation. Cysteine residues are indicated in bold and their pairing by black lines. The secondary structure elements of the protein are indicated below the sequence in yellow, red, and green for β-strand, α-helix, and coil, respectively. The N-terminal domain (Pca1 to Glu42) is hydrophobic and adopts a β1-α1-α2-β2 fold (β1, Thr15-Val16; α1, Ala18-Val27; α2, Gly29-Arg35; β2, Arg39-Arg40). The C-terminal domain adopts a β-defensin-like fold (Cys57-Cys87, Cys64-Cys82, Cys68-Cys88), a four-stranded antiparallel β-sheet (β3, Ser56-Cys57; β4, Gly62-Arg65; β5, His71-Asp75; β6, Tyr86-Pro91), and an α-helix (α3, Trp76-Cys82). (B) Superimposition of the 10 models representative of *Cg*-BigDef1[1–93] solution structure with a root mean square deviation (RMSD) at 0.567 Å. (C) Electrostatic positive (blue) and negative (red) areas calculated at the Connolly surface by the use of the Adaptive Poisson-Boltzmann Solver (APBS) (54). (D) Hydrophobic (yellow) and hydrophilic (green) potential areas calculated at the Connolly surface by the use of the Platinum (55).

The 3D structures of *Cg*-BigDef1[1–93] and *Cg*-BigDef1[44–93] were determined by NMR spectroscopy (Tables S1 and S2). *Cg*-BigDef1[1–93] is composed of two distinct globular domains connected by a flexible linker (Fig. 3). The N-terminal domain (Pca1 to Glu42) is hydrophobic and adopts a β1-α1-α2-β2 fold. A hydrophobic core composed of residues Tyr11, Val16, Val20, Leu24, Leu33, and Ile38 stabilizes this highly compact fold. The C-terminal domain adopts a β-defensin-like fold with a cysteine pairing identical to that of β-defensins (C1-C5, C2-C4, and C3-C6), although the cysteine spacings differ; it also displays a typical four-stranded antiparallel β-sheet and an α-helix (Fig. 3). The linker is composed of 10 residues (Asn43 to His52) and is located at the interface of the two globular domains (strong polar contacts are formed during the structure calculation as follows: Arg49/Thr15, Asp51/Ser54, His52/Asp53, Arg65/Asp51, and His71/Asn43). This allows *Cg*-BigDef1[1–93] to be highly compact in solution.

10.1128/mBio.01821-19.7 TABLE S1 NMR constraints and structural statistics for *Cg*-BigDef1[1–93]. Download Table S1, DOCX file, 0.01 MB. Copyright © 2019 Loth et al. 2019 Loth et al. This content is distributed under the terms of the Creative Commons Attribution 4.0 International license.

We found that the fold of *Cg*-BigDef1[44–93] is identical to that of the C-terminal domain of *Cg*-BigDef1[1–93] except for the first nine residues (44 to 52), *viz.*, the linker, as the network of polar contacts cannot be formed in the absence of the N-terminal domain (Fig. S3 and S4). The surface of *Cg*-BigDef1[1–93] is mainly hydrophobic, with no amphipathic properties. Indeed, all the positively charged residues (Arg35, Arg39, Arg40, and Arg41) of the N-terminal domain are exposed at the surface. Arg39 is surrounded by four tyrosine residues (Tyr28, Tyr31, Tyr34, and Tyr36) forming a hydrophilic patch. Arg40 and Arg41 create an extended hydrophilic belt at the interface between the β-sheet of the hydrophobic domain, the linker, and the C-terminal domain (Ser10, Thr15, Arg40, Arg41, Glu42, Arg47, Arg49, Ser50, Asp51, His52, Asp53, Thr67, Ser70, His71, and Arg92). The C-terminal domain (net charge, +3) exhibits three positively charged residues (Arg61, Arg89, and Arg91) and two negatively charged acidic residues (Asp53 and Asp80) at the surface, the other charged residues being buried. Overall, the N-terminal domain does not alter the fold of the conserved β-defensin-like domain but confers hydrophobic properties to this molecule, which is otherwise mainly hydrophilic.

10.1128/mBio.01821-19.4 FIG S3 *Cg*-BigDef1[44–93] global fold and comparison with that of *Cg*-BigDef1[1–93]. (A) *Cg*-BigDef1[44–93] (black) and *Cg*-BigDef1[1–93] (magenta) primary structures. Cysteine residues are indicated in bold, and their pairings are indicated by black lines. (B) Overlay of results of ^15^N-sofast-HMQC analyses of *Cg*-BigDef1[44–93] (black) and *Cg*-BigDef1[1–93] (magenta). The assignment of *Cg*-BigDef1[1–93] is reported. Both peptides were highly structured as shown by a good dispersion of the amide chemical shifts in their ^1^H NMR and sofast-HMQC spectra. (C) Superimposition of the 10 models representative of *Cg*-BigDef1[44–93] solution structure. (D) Structure alignment of *Cg*-BigDef1[44–93] (black) and *Cg*-BigDef1[1–93] (magenta). Download FIG S3, DOCX file, 0.3 MB. Copyright © 2019 Loth et al. 2019 Loth et al. This content is distributed under the terms of the Creative Commons Attribution 4.0 International license.

### *Cg-*BigDef1 has broad range bactericidal activity.

*Cg-*BigDef1 antimicrobial activities were tested at 150 mM NaCl and 400 mM NaCl, physiological salt concentrations for humans and marine bacteria, respectively (Table 2). *Cg-*BigDef1 was active against a range of reference, environmental, oyster, and human clinical strains, with various profiles of susceptibility to antibiotics (see Table S3 in the supplemental material). The lowest minimal inhibitory concentrations (MICs) were observed against Gram-positive strains (Table 2). All S. aureus strains tested, including methicillin-resistant S. aureus (MRSA) clinical isolates from cystic fibrosis (CF) and non-CF patients, were susceptible to *Cg-*BigDef1 (MICs in the 1.25 to 5 μM range). *Vibrio* isolates, including V. tasmaniensis and V. crassostreae strains pathogenic for oysters, were inhibited in the 1.25 to 10 μM range. Full inhibition of human clinical isolates of *Pseudomonas* and *Burkholderia* was not reached at the highest concentration tested. A bactericidal effect against most susceptible strains was determined with minimum bactericidal concentrations (MBCs) in the range of 0.6 to 10 μM (Table 2).

**TABLE 2:** Antimicrobial activities of the full *Cg-*BigDef1 and its isolated domains^*a*^

Strain	Source	[NaCl] (mM)	*Cg*-BigDef1[1–93]		*Cg*-BigDef1[1–42]		*Cg*-BigDef1[44–93]	
			MIC (μM)	MBC (μM)	MIC (μM)	MBC (μM)	MIC (μM)	MBC (μM)
Gram-negative bacteria								
Aliivibrio fischeri 7P_21	Env	400	>10	>10	>10	>10	>10	>10
Burkholderia multivorans 12/11/13-B-2333	Clin/h	150	>10	>10	>10	>10	>10	>10
Escherichia coli MC4100	Ref	150	>10	>10	>10	>10	>10	>10
Pseudomonas aeruginosa ATCC 9027	Ref	150	>10	>10	>10	>10	>10	>10
Pseudomonas aeruginosa (Pa25) 13/07/11-B-3003	Clin/h	150	>10	>10	>10	>10	>10	>10
Pseudomonas aeruginosa (Pa02) 12/07/11-B-2011	Clin/h	150	>10	>10	>10	>10	>10	>10
Vibrio tasmaniensis LGP32	Clin/o	400	10	>10	>10	>10	>10	>10
Vibrio tasmaniensis 3T8_11	Clin/o	400	10	10	20	>10	>10	>10
Vibrio tasmaniensis 7G7_3	Clin/o	400	5	20	>10	>10	>10	>10
Vibrio crassostreae 7T4_12	Clin/o	400	5	>10	20	>10	>10	>10
Vibrio crassostreae 7F5_29	Clin/o	400	1.25	1.25	10	>10	5	10
Vibrio orientalis 8F5_42	Env	400	10	>10	20	>10	>10	>10
Vibrio breoganii 7F1_16	Clin/o	400	10	>10	20	>10	20	>10
Vibrio harveyi 7G5_1	Clin/o	400	>10	>10	>10	>10	>10	>10

Gram-positive bacteria								
Corynebacterium stationis CIP 101282	Ref	400	0.15	0.6	2.5	10	2.5	10
Microbacterium maritypicum CIP 105733^T^	Ref	400	>10	>10	20	>10	10	>10
Micrococcus luteus CIP 53.45	Ref	150	0.3	1.25	2.5	10	10	>10
Staphylococcus aureus (MRSA) strain 7877	Clin/h	150	2.5	5	>10	>10	>10	>10
Staphylococcus aureus (MRSA) strain 53863	Clin/h	150	2.5	>10	>10	>10	>10	>10
Staphylococcus aureus (MRSA) 31/01/14-B-5284	Clin/h	150	1.25	>10	>10	>10	>10	>10
Staphylococcus aureus (MRSA, GISA) 24/11/08-B-1347	Clin/h	150	2.5	>10	>10	>10	>10	>10
Staphylococcus aureus (MSSA) 07/02/14-B-5264	Clin/h	150	5	10	>10	>10	>10	>10
Staphylococcus aureus Newman	Ref	150	2.5	>10	>10	>10	>10	>10
Staphylococcus aureus SG511	Ref	150	1.25	>10	10	>10	>10	>10

a MIC values (reported in micromoles per liter [μM]) refer to the minimal concentration required to achieve 100% growth inhibition. MBC values (reported in micromoles per liter) refer to the minimal concentration required to kill 100% of bacteria. The NaCl concentrations at which assays were performed are indicated in millimoles per liter (mM). The origin of the clinical and environmental isolates is specified in Table S3. Env, environmental isolate; Clin, clinical isolate from either human (Clin/h) or oyster (Clin/o); Ref, reference strain; NT, not tested; CIP, Collection de l’Institut Pasteur; ATCC, American Type Culture Collection; MSSA, methicillin-susceptible Staphylococcus aureus; MRSA, methicillin-resistant Staphylococcus aureus; GISA, glycopeptide-intermediate Staphylococcus aureus.

10.1128/mBio.01821-19.8 TABLE S2 NMR constraints and structural statistics for *Cg*-BigDef1[44–93]. Download Table S2, DOCX file, 0.01 MB. Copyright © 2019 Loth et al. 2019 Loth et al. This content is distributed under the terms of the Creative Commons Attribution 4.0 International license.

10.1128/mBio.01821-19.9 TABLE S3 Strains and media. Download Table S3, DOCX file, 0.03 MB. Copyright © 2019 Loth et al. 2019 Loth et al. This content is distributed under the terms of the Creative Commons Attribution 4.0 International license.

### Covalent association of *Cg-*BigDef1 domains is essential for salt-stable antimicrobial activity.

The separate domains were markedly less bactericidal than full-length *Cg-*BigDef1 (Table 2). Synergies between the two domains were therefore measured by exposing bacteria to *Cg*-BigDef1[1–42] and *Cg*-BigDef1[44–93] simultaneously or to *Cg*-BigDef1[1–93]. The separate domains acted synergistically against both Gram-positive and Gram-negative strains, with fractional inhibitory concentration (FIC) index values in the 0.625 to 0.740 range (i.e., <1) (Table 3). Strong synergy was observed when domains were linked covalently (FIC values in the 0.067 to 0.154 range; <0.5) (Table 3). This shows that the antimicrobial activity of the β-defensin domain *Cg*-BigDef1[44–93] is dependent on a covalent association with the N-terminal *Cg*-BigDef1[1–42] domain.

**TABLE 3:** FIC index values of the N- and C-terminal domains of *Cg-*BigDef1^*a*^

Strain	FIC index	
	Covalently linked domains	Separate domains
Staphylococcus aureus SG511	0.156	0.625
Corynebacterium stationis CIP 101282	0.067	0.740
Micrococcus luteus CIP 53.45	0.135	0.750
Vibrio crassostreae 7F5_29	0.125	0.750

a The synergies of the N- and C-terminal domains were measured as described previously (51) by incubating either both domains or the full-length *Cg*-BigDef1 (i.e., covalently linked domains) with bacterial suspensions of 4 strains displaying the lowest MICs for *Cg*-BigDef1[1–93]. Results are expressed as FIC index values according to the following formula: FIC = (N-ter)/MIC_N-ter_ + (C-ter)/MIC_C-ter_, where MIC_N-ter_ and MIC_C-ter_ are the MICs of the N- and C-terminal domains tested alone and (N-ter) and (C-ter) are the MICs of the two peptides tested in combination. FIC index values are interpreted as follows: <0.5, strong synergy; 0.5 to 1, synergy; 1 to 2: additive effect; 2, no effect; >2, antagonism.

**FIG 4:**
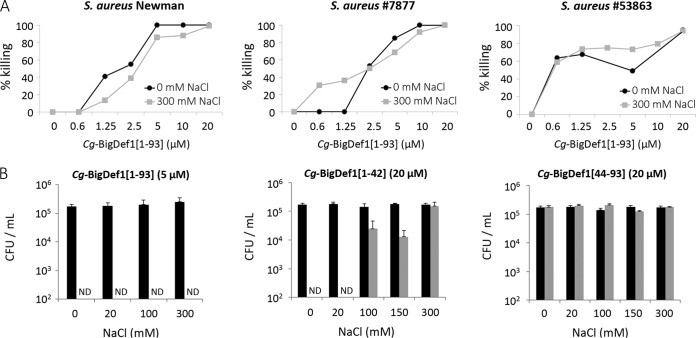
*Cg-*BigDef1 antimicrobial activity is stable at high salt concentrations. (A) Effect of NaCl (0 and 300 mM) on the bactericidal activity of various concentrations of *Cg*-BigDef1[1–93] against laboratory S. aureus strain Newman and two cystic fibrosis clinical isolates of S. aureus (strain 7877 and strain 53863). (B) Effect of increasing NaCl concentrations on the antibacterial activity of *Cg*-BigDef1[1–93] at 5 μM, *Cg*-BigDef1[1–42] at 20 μM, and *Cg*-BigDef1[44–93] at 20 μM against S. aureus Newman. Bacterial cells were incubated with the indicated peptides (gray bars) or the corresponding solvents (black bars) in killing buffer (KB) in the presence of 0, 20, 100, or 300 mM NaCl. After 2 h, bacterial suspensions were serially diluted with phosphate-buffered saline (PBS) and aliquots were streaked on Luria-Bertani (LB) agar plates and incubated for 24 h at 37°C. Bactericidal effects were monitored by counting the bacterial CFU on LB agar plates and expressed either as percent killing compared with that seen with treatment without the antimicrobial peptide (A) or as CFU counts per milliliter (B). ND, not detected (<100 CFU per milliliter).

Next, we focused our subsequent studies on S. aureus, which was highly sensitive to *Cg-*BigDef1 activity. We showed that *Cg*-BigDef1[1–93] was bactericidal up to 300 mM NaCl against both the laboratory strain S. aureus Newman and the multidrug-resistant S. aureus clinical isolates 7877 and 53863 (Fig. 4A). At 5 μM, *Cg*-BigDef1[1–93] was bactericidal against S. aureus Newman in the range of 0 to 300 mM NaCl whereas NaCl by itself had no effect on bacterial growth (Fig. 4B). A bactericidal effect was recorded for *Cg*-BigDef1[1–42] in the absence of NaCl as a consequence of raising the peptide concentration to 20 μM. This effect was lost at concentrations over 100 mM NaCl (Fig. 4B). No bactericidal activity was recorded for 20 μM *Cg*-BigDef1[44–93] in the NaCl concentration range of 0 to 300 mM (Fig. 4B). Therefore, unlike its separate domains, *Cg*-BigDef1[1–93] shows salt-stable bactericidal activity.

### *Cg-*BigDef1 entraps bacteria in nanonets without inducing membrane permeabilization.

**FIG 5:**
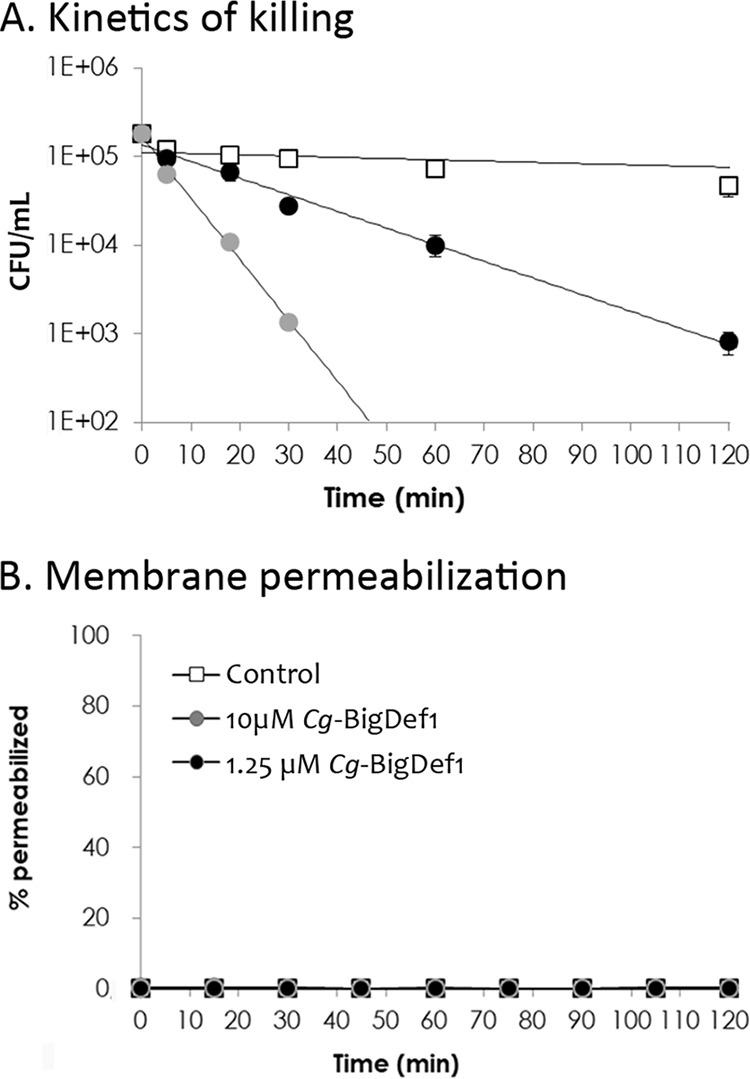
*Cg-*BigDef1 bactericidal activity is not coupled to membrane permeabilization. (A) The time course of *Cg-*BigDef1 killing of S. aureus SG511 was measured over 120 min at two *Cg-*BigDef1 concentrations (1.25 μM [black symbols] and 10 μM [gray symbols]). In a control experiment, *Cg-*BigDef1 was replaced by an equal volume of water (white squares). At time zero, cultures were adjusted to 10^5^ CFU/ml. CFU were then counted by plating at 10, 20, 30, 60, and 120 min. In this assay, the limit of detection was 100 CFU/ml. (B) Membrane permeabilization of S. aureus SG511 was measured by the Sytox green assay. Bacteria were exposed to 1.25 or 10 μM *Cg-*BigDef1 or an equal volume of water (control). Data are represented as percentages of permeabilized bacterial cells relative to complete cell lysis with Triton X-100 as a positive control. In both assays, standard deviations were calculated using results from three independent experiments.

**FIG 6:**
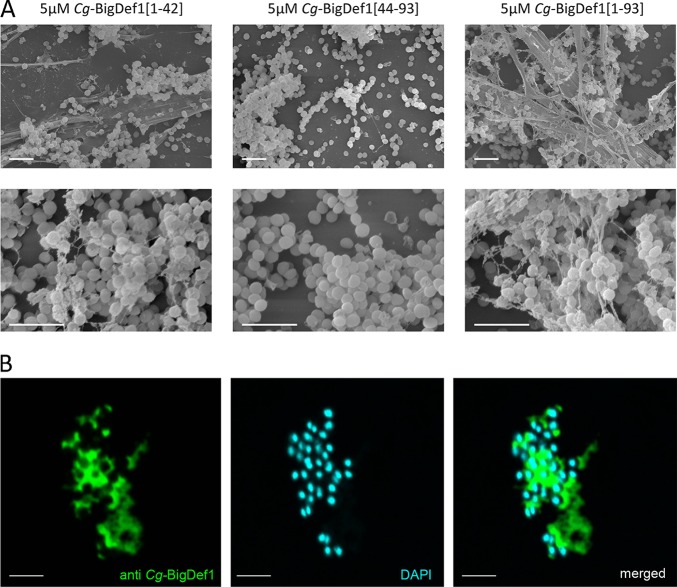
The N-terminal domain drives bacterially triggered assembly of *Cg-*BigDef1 into nanonets. (A) SEM observation of *Cg-*BigDef1 nanonets. Large and branched fibers entrapping S. aureus SG511 were observed in S. aureus samples subjected to 24 h of exposure to 5 μM *Cg*-BigDef1[1–93] or *Cg*-BigDef1[1–42] but not 5 μM *Cg*-BigDef1[44*–*93], which is indistinguishable from the no-peptide control results (not shown). (B) Immune staining of *Cg-*BigDef1 nanonets in contact with S. aureus SG511. Confocal microscopy images were acquired after 30 min of contact between bacteria and peptides. DNA was stained with DAPI (blue), and *Cg-*BigDef1 was stained with polyclonal antibodies (anti-*Cg*-BigDef1) revealed with a secondary antibody coupled to Alexa Fluor 488 (green). Merged images show that space occupied by S. aureus cells is left empty by *Cg-*BigDef1 nanonets. Bars represent 3 μm.

We further explored the mechanism of action of *Cg-*BigDef1 on S. aureus*. Cg*-BigDef1[1–93] killed cells of the SG511 reference strain at 1.25 μM, with a 2-log reduction in colony-forming unit (CFU) counts after 2 h. At 10 μM, no CFU could be counted after 60 min (Fig. 5A) and no membrane permeabilization was detected (Fig. 5B). Therefore, *Cg-*BigDef1 bactericidal activity is independent of membrane permeabilization in S. aureus. Remarkably, by observing *Cg-*BigDef1-treated S. aureus by scanning electron microscopy (SEM), we found that *Cg*-BigDef1[1–93] undergoes intense fibrilar aggregation upon contact with bacteria. Bacterial cells were entrapped in highly branched nanonets and/or covered with large fibers that adhered to the bacterial surfaces when incubated with 5 μM *Cg*-BigDef1[1–93] (Fig. 6A). Such structures were not observed in the absence of bacteria (data not shown), which indicates that bacteria play an essential role in triggering their assembly. Similar structures were observed when bacteria were incubated with 5 μM *Cg*-BigDef1[1–42] but not when they were incubated with β-defensin-like *Cg*-BigDef1[44–93] (5 μM) (Fig. 6A). This supramolecular assembly is consistent with the loss of solubility of *Cg*-BigDef1[1–93] and *Cg*-BigDef1[1–42] after 30 min of contact with S. aureus (Fig. S5 in the supplemental material). By using polyclonal antibodies raised against *Cg*-BigDef1[1–93], we observed intense immune staining of large areas surrounding S. aureus under conditions of incubation with *Cg*-BigDef1[1–93] (5 μM) (Fig. 6B). This showed that the observed fibers represented *Cg*-BigDef1[1–93] nanonets. Confocal sections revealed the absence of immune staining at positions occupied by bacterial cells, showing that *Cg*-BigDef1[1–93] did not enter bacteria intracellular space, at least during 30 min of contact (Fig. 6B).

10.1128/mBio.01821-19.5 FIG S4 Accurate mass determination of fragments of tryptic peptides 50 to 92 from *Cg*-BigDef1[1–93] and *Cg*-BigDef1[44–93]. The tryptic digest of *Cg*-BigDef1[1–93], obtained under nonreducing conditions in the presence of 1% RapiGest surfactant, was analyzed by LC-ESI-MS, using a high-resolution Q-Exactive Orbitrap analyzer, and the resulting data file was processed in order to translate the observed multicharged peptide ions (A) into the corresponding monoisotopic mass of the compounds at the origin of these ions (B). On the basis of their masses, two observed values likely correspond to tryptic peptides 50 to 93 (observed mass, 5,188.093 Da) and fragments consisting of to tryptic peptides 50 to 92 (observed mass, 5,031.977 Da), with all six cysteine residues involved in disulfide bonds. Additionally, two compounds accompanied the peptide ions of the fragment consisting of tryptic peptides 50 to 92 and were resolved into masses of 5,049.985 Da and 5,067.999 Da, showing an addition of 18 Da in each case. These two ions likely correspond to the result of the hydrolysis by trypsin of either one or two amide bonds after the available arginine in this part of the protein sequence, as shown in the text inserted in panel B. (C) The same process applied to *Cg*-BigDef1[44–93] resulted in a similar spectrum. Download FIG S4, DOCX file, 0.1 MB. Copyright © 2019 Loth et al. 2019 Loth et al. This content is distributed under the terms of the Creative Commons Attribution 4.0 International license.

10.1128/mBio.01821-19.6 FIG S5 Loss of *Cg*-BigDef1[1–93] and *Cg*-BigDef1[1–42] solubility after 30 min of contact with S. aureus*. Cg*-BigDef1[1–93], *Cg*-BigDef1[1–42], or *Cg*-BigDef1[43-93] was added (5 μM final concentration) to 200 μl of a solution of Tris-HCl (100 mM; pH 8), CaCl_2_ (1 mM), and NaCl (150 mM) containing a suspension of bacteria (optical density [OD] of 0.1). After 30 min at 20°C, bacteria were pelleted by centrifugation and the supernatant was collected. In control tubes, peptides were added to 200 μl of buffer only. After acidification by TFA treatment, 90 μl of supernatant was injected in an HPLC instrument and the peak areas (OD at 225 nm) for the present *Cg-*BigDef1 peptide were measured. The graphs at the top show the peak areas from samples of bacteria in proportion to the area obtained for controls (buffer plus peptides). The results indicated that the peptides had retained their entire solubility in buffer. Download FIG S5, DOCX file, 0.04 MB. Copyright © 2019 Loth et al. 2019 Loth et al. This content is distributed under the terms of the Creative Commons Attribution 4.0 International license.

### *Cg-*BigDef1 is neither cytotoxic nor proinflammatory toward mammalian cells.

**FIG 7:**
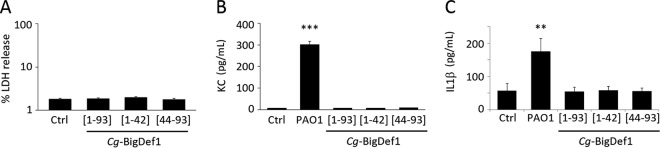
*Cg-*BigDef1 is neither cytotoxic nor proinflammatory toward eukaryotic cells. (A) Cytotoxicity assay. NCI-H292 cells were incubated with 5 μM *Cg*-BigDef1[1–93], 20 μM *Cg*-BigDef1[1–42], or 20 μM *Cg*-BigDef1[44–93]. The release of lactate dehydrogenase (LDH) by the cells was measured after 24 h using the CytoTox 96 nonradioactive cytotoxicity assay (Promega). Triton X-100 (1%) was used as a positive control (100% LDH release). Untreated cells were used as a negative control (Ctrl). Data are expressed as a percentage of the total LDH release compared to cells treated with Triton X-100 (for more details, see reference 22). (B and C) Proinflammatory assays. Macrophage cells (cell line J774) were incubated with peptides at the same concentrations. Secretion of KC (B) and IL-1β (C) was measured after 24 h using DuoSet ELISA kits as previously described (24). As a positive control for cytokine/chemokine secretion, J774 cells were infected with P. aeruginosa PAO1 (multiplicity of infection [MOI] 1:1).

We finally examined *Cg-*BigDef1 and its separate domains for potential toxic and/or proinflammatory effects on eukaryotic cells. None of the three peptides were toxic toward bronchial epithelial cell line IB3 isolated from CF patients, as they did not induce any detectable release of lactate dehydrogenase compared to the 100% release seen in the Triton X-100 positive control (Fig. 7A). Moreover, they did not induce a proinflammatory response in mouse alveolar macrophage cell line J774. Indeed, none of the peptides triggered secretion of keratinocyte-derived protein chemokine (KC) (Fig. 7B), the mouse homologue of interleukin-8 (IL-8) known to induce neutrophil chemotactic activity (23), leading to the accumulation of these cells in the site of infection. Similar results were obtained for IL-1β (Fig. 7C), which promotes bacterial killing by alveolar macrophages (24). In contrast, infection of J774 cells by Pseudomonas aeruginosa PAO1 (positive control) led to high levels of secretion of both KC and IL-1β (Fig. 7B and C).

## DISCUSSION

During evolution, the activity of β-defensins has broadened beyond direct antimicrobial action such that in mammals, some peptides have adopted a series of additional functions in immunity (e.g., immune modulation, chemoattraction) and reproduction; they are currently considered to represent multifunctional HDPs rather than solely AMPs (3, 25). Here, by studying big defensins, the ancestors of β-defensins, we uncovered unique physicochemical properties essential for their ancestral antimicrobial activity. These properties have been lost during evolution toward β-defensins but have been preserved in a limited number of marine species.

We found that oyster *Cg-*BigDef1 has conserved salt-stable antimicrobial activity. Indeed, *Cg*-BigDef1 showed a broad spectrum of activity in the 1 to 10 μM range, even at high salt concentrations. It was active at up to 300 mM NaCl against human pathogens and 400 mM NaCl against oyster pathogens (marine microorganisms). The two domains of *Cg*-BigDef1 did not display complementary spectra of activities, as initially proposed for the horseshoe crab *Tt-*BigDef (10). Instead, they were highly synergistic, highlighting the importance of their covalent association for the whole peptide activity. NMR data revealed that oyster *Cg-*BigDef1, like horseshoe crab *Tt-*BigDef (26), possesses two structural domains. The hydrophobic N-terminal domain adopts a unique globular fold, and the C-terminal domain adopts a β-defensin-like conformation. The two domains are in close contact, giving rise to a very compact 3D structure in solution. While cationic and anionic patches are displayed at the surface of *Cg-*BigDef1, the overall structure is mainly hydrophobic and does not display the typical amphipathic structure of cationic AMPs (27). This has important consequences for *Cg-*BigDef1 interactions with bacteria, which were shown to require the hydrophobic N-terminal domain and are not impaired at high salt concentrations. This suggests that *Cg-*BigDef1 interacts with bacterial membranes through hydrophobic interactions rather than electrostatic interactions.

**FIG 8:**
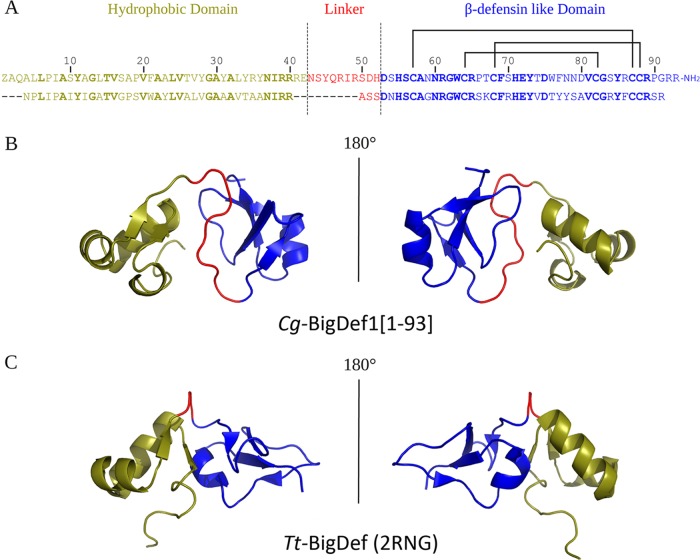
*Cg*-BigDef1[1–93] and *Tt*-BigDef 3D structure comparison. The hydrophobic domain, the linker, and the β-defensin-like domain are colored in deep olive, red, and blue, respectively. (A) Alignment of *Cg*-BigDef1[1–93] (top) and *Tt*-BigDef (bottom) primary sequences. Conserved residues are indicated in bold. (B) 3D structure (cartoon representation) of *Cg*-BigDef1[1–93] (PDB entry 6QBL). (C) 3D structure (cartoon representation) of *Tt*-BigDef (PDB entry 2RNG).

The 3D structure of *Cg-*BigDef1 is only the second from the big defensin family. It differs from *Tt-*BigDef by exhibiting a longer linker and a different orientation of the N- and C-terminal domains (Fig. 8). By modifying the orientation of the domains, we found that linkers drastically modified the surface properties of big defensins. Indeed, *Tt-*BigDef is amphipathic whereas *Cg-*BigDef1 is hydrophobic. Whether this orientation modifies peptide activity and/or stability with respect to salts remains to be established. Indeed, information is still missing on the activities of *Tt-*BigDef and other big defensins, precluding further interpretations. In a micellar (membrane-like) environment, the N-terminal domain of *Tt-*BigDef adopts a single α-helix structure, which penetrates into micelles; it was hypothesized that insertion of this helix into target membranes may be involved in the *Tt*-BigDef antimicrobial activity (11). According to our results, such a membrane activity is not responsible for *Cg-*BigDef1 activity, although it could confer antimicrobial activity to the *Cg-*BigDef1 N-terminal domain at a high concentration (20 μM).

The ancestral N-terminal domain of *Cg-*BigDef1 was shown to drive bacterially triggered assembly of *Cg-*BigDef1 into nanonets, while *Cg*-BigDef1 appeared highly soluble and monomeric in solution. These nanonets entrapped and killed S. aureus. The N-terminal domain alone also produced nanonets but did not kill S. aureus. This suggests that *Cg-*BigDef1 antimicrobial activity is carried by the β-defensin-like domain but requires the N-terminal domain to promote close contact with bacteria. The mechanism by which the N-terminal drives nanonet formation remains to be characterized. In human α-defensin 6 (HD6), which self-assembles into elongated fibrils and agglutinates bacteria (28), hydrophobic amino acids play a key role (29). For *Cg-*BigDef1 to achieve self-assembly, the amino acids of the hydrophobic core need to be exposed through a partial unfolding process or conformational change. Micelles of dodecylphosphocholine induced such a conformational change of *Tt*-BigDef N-terminal domain (26). However, no report has been published on the possibility that *Tt*-BigDef can form nanonets. Surprisingly, whereas the big defensin N-terminal domain was lost during evolution toward β-defensins, human BD1 (hBD1) was recently shown to form nanonets in its reduced form (30), which is the active form of hBD1 in the colonic environment (31). This suggests that nanonet formation may have emerged independently in diverse families of AMPs, highlighting a neglected function of AMPs, which can entrap bacteria and prevent subsequent host colonization (29, 30). Whether all AMPs that self-assemble into nanonets form highly ordered structures, as observed for HD6 *in vitro* (29), or protein aggregates remains unknown. According to the results of the HD6 study, nanonets are highly distinct from β-amyloid fibrils, although assembly benefits from hydrophobic interactions in both cases. The mechanisms triggering nanonet assembly in structurally unrelated peptide families remain largely unknown. It has been proposed for HD6 that bacterial surface proteins provide a nucleation site for peptide self-assembly (28).

We showed here that oyster *Cg-*BigDef1 exerts antimicrobial activity, even at the high physiological salt concentrations of its marine host. This salt-stable antimicrobial activity is conferred by the hydrophobic properties of the ancestral N-terminal domain lost in β-defensins. The ability to osmoregulate, when present, is not as efficient in chelicerates and mollusks as in vertebrates, and oysters themselves are osmoconformers (32, 33). Consequently, the hemolymph concentration of Na^+^ and Cl^−^ ions is very close to their concentration in seawater. We believe that such strong selection pressures imposed by marine environments could have preserved ancestral big defensins from evolving toward (salt-sensitive) β-defensins in organisms with poor control of their blood osmolarity. In contrast, an evolutionary cost may have accelerated big defensin molecular evolution in species that osmoregulate their body fluids and have more efficient immune systems, such as vertebrates, or that live in freshwater. The only big defensin gene present in a freshwater organism (mussel) remains a puzzling exception (21); this gene may have evolved toward other functions after transition of mussel species from seawater to freshwater.

Interestingly, we found that *Cg*-BigDef1 is active against methicillin-resistant S. aureus at micromolar concentrations and under high salt conditions without being cytotoxic or proinflammatory toward mammalian cells. This is important, as small peptides that self-assemble can be toxic to mammals (34). MRSA is a major cause of mortality due to antibiotic-resistant infections (35). As antimicrobial resistance threatens human health and the core of modern medicine, the WHO is calling upon the design of new drugs active against multiresistant bacteria (36). On the basis of the complementary action of two domains, the mechanism of action of the *Cg*-BigDef1 complex would be an interesting trait to explore. We can indeed speculate that grafting the ancient N-terminal domain to vertebrate β-defensin would confer high stability with respect to salt and increase the spectrum of activity, as shown here for the two domains of *Cg*-BigDef1. This reconstruction of ancient big defensins not only may have synergistic effects, as reported for other AMP combinations (37) but may also confer a great advantage, as resistance rises at lower rates when combinations of AMPs are used (38). Considering the current interest in AMPs (39), we believe that analysis of ancestral big defensins can inspire the design of novel antimicrobials that will be efficient at physiological salt concentrations but that will also be applicable for treatment of diseases involving salt imbalance or for which salt treatment is used, such as cystic fibrosis (40).

## MATERIALS AND METHODS

### Database searches and sequence analysis.

Sequences containing the β-defensin domain were collected from publicly accessible databases and were used for the search of homologous sequences in both annotated and nonannotated databases (tBLASTx at NCBI). All obtained sequences were manually inspected and translated using the ExPASy Translate Tool (http://web.expasy.org/translate/). Predictions of signal peptides and furin-like cleavage sites were performed with the ProP 1.0 Server (http://www.cbs.dtu.dk/services/ProP/). Multiple alignments of the deduced amino acid sequences were generated using the MAFFT program (https://mafft.cbrc.jp/alignment/server/).

### Chemical synthesis.

Peptides were synthesized by solid-phase peptide synthesis on a Prelude peptide synthesizer (Gyros-Protein Technologies) using standard Fmoc/*t*Bu chemistry at the 25-μmol scale starting from a Tentagel resin equipped with a Rink’s amide linker and including automated introduction of the *N*-2-hydroxy-5-nitrobenzyl (Hnb) group trough on resin reductive amination (22). *Cg-*BigDef1 was obtained under standard conditions (19) through native chemical ligation (NCL) of an N-terminal cysteinyl peptide segment (*Cg*-BigDef1[57–93]) and the crypto-thioester *Cg*-BigDef1[1–56]-(Hnb)Cys(S*t*Bu). After high-performance liquid chromatography (HPLC) purification, the reduced form was engaged into thermodynamically controlled oxidative folding to form the three disulfide bridges, using already-described protocols (41, 42). Purification by C4 RP-HPLC afforded the pure oxidized form of *Cg-*BigDef1 (see supplemental material).

### Online liquid chromatography coupled to electrospray ionization tandem mass spectrometry (LC-ESI-MS/MS).

**(i) Chemicals.** MilliQ water (Merck Millipore, Billerica, MA) was used. LC-MS-grade formic acid (FA), trifluoroacetic acid (TFA), and acetonitrile (MeCN) were from Carlo-Erba (Val de Reuil, France). Reagent-grade chemicals for protein preparation and proline endopeptidase (E1411) were from Sigma-Aldrich (St. Louis, MO). Sequencing-grade modified trypsin and GluC were from Promega (Madison, WI). RapiGest surfactant (SF) was from Waters (Milford, MA).

**(ii) Big defensin enzymatic digestion.** Synthetic peptides were digested (overnight, 37°C) by adding RapiGest SF (0.1% to 1%) and protease (1:10 ratio). Following acidification, the digest was dried using a speed vacuum apparatus (FreeZone Plus 2.5-liter freeze-dry system; Labconco, Kansas City, MO, USA) and suspended in 2% MeCN–0.1% TFA (vol/vol).

**(iii) Peptide analysis by LC-ESI-MS/MS.** An Agilent HPLC HP-1290 system (Agilent Technologies, Santa Clara, CA) on-line coupled to a Q-Exactive Orbitrap mass spectrometer (Thermo Scientific, Bremen, Germany) was used. Separation was performed on an Accucore C_18_ column (Thermo Scientific) (2.1 mm by 150 mm) at 35°C with a flow rate of 350 μl/min. Solvent A was 0.1% FA–water and solvent B was 0.1% FA–MeCN, and the gradient was from 2% solvent B to 15% in 26 min and then to 62% in 34 min. Typically, volumes of 0.5 to 2 μg of peptide were injected. The Q-Exactive Orbitrap mass spectrometer was operated as previously published (43, 44) except for the automatic lock mass function (enabled on the ion at *m/z* 593.15761).

### NMR experiments and calculation of structures.

*Cg*-BigDef1[44–93] and *Cg*-BigDef1[1–93] were dissolved in H_2_O:D_2_O (9:1 ratio) at concentrations of 1.5 mM and 1.0 mM, respectively. pH was adjusted to 4.6 for both samples. 2D ^1^H nuclear Overhauser effect spectroscopy (NOESY), 2D ^1^H total correlation spectroscopy (TOCSY), band-selective optimized flip angle short transient–heteronuclear multiple quantum coherence (sofast-HMQC) (45) (^15^N natural abundance), and ^13^C-HSQC (^13^C natural abundance) were performed at 298 K on an Avance III HD Bruker 700 MHz spectrometer equipped with a cryoprobe. ^1^H chemical shifts were referenced to the water signal (4.77 ppm at 298 K). NMR data were processed using Bruker’s Topspin 3.2 and analyzed with CCPNMR software (version 2.2.2) (46). Structures for both proteins were calculated using the Crystallography and NMR system (CNS) suite (47, 48) through the use of ARIA2 automatic assignment software (version 2.3) (49) with NOE derived distances and hydrogen bonds and three ambiguous disulfide bridges. For *Cg*-BigDef1[44–93], backbone dihedral angle restraints were added (determined with the DANGLE program [50]). See detailed protocol in Text S1 in the supplemental material.

10.1128/mBio.01821-19.1 TEXT S1 NMR and structure calculations. Download Text S1, DOCX file, 0.03 MB. Copyright © 2019 Loth et al. 2019 Loth et al. This content is distributed under the terms of the Creative Commons Attribution 4.0 International license.

### Strains and media.

Strains and media are listed in Table S3 in the supplemental material. Marine strains from the genera *Aliivibrio*, *Corynebacterium*, *Microbacterium*, and *Vibrio* were grown at 20°C in liquid Zobell 1/3 medium. Other strains were grown at 30°C in liquid Poor broth (PB) medium (Table S3). Zobell or Luria-Bertani (LB) agar plates were used as solid media.

### Antimicrobial assays.

**(i) Determination of MICs and minimal bactericidal concentrations (MBCs).** For antibacterial assays, MICs and MBCs were determined by the liquid growth inhibition assay previously described (51). For antifungal assays, inhibition of spore germination was monitored by a previously described liquid growth inhibition assay (52). Synergies (FIC index) between peptides were measured as previously described (51).

**(ii) Bactericidal assays and kinetics of killing.** Bacteria (10^5^ to 10^6^ CFU/ml) were incubated at 37°C with or without peptide in killing buffer (KB; 0.1 M Tris [pH 8.0], 1 mM CaCl_2_) supplemented with 0.1% fatty acid-free bovine serum albumin and 0 to 300mM NaCl. CFU counts were performed by plating on LB agar plates at the indicated times. Data are expressed either as CFU per milliliter or as a percentages of killing compared to untreated samples. Tests were performed in triplicate, and mean values were calculated.

### Membrane permeabilization assay.

Stationary-phase cultures of S. aureus SG511 grown at 37°C in LB medium were washed and resuspended at 10^9^ CFU/ml in 10 mM phosphate-buffered saline (PBS), supplemented with 138 mM NaCl and 2.7 mM KCl; pH was adjusted to 7.4 before addition of 1 μM Sytox green. Bacteria (100 μl) were dispensed into 96-well microtiter plates containing 10 μl *Cg-*BigDef1 (10 μM or 1.25 μM final concentration), water (negative control), or Triton X-100 (0.1% final concentration; positive control). Fluorescence was measured every 5 min over 4 to 6 h (λ excitation [λex] = 480 nm; λ emission [λem] = 550 nm) at 20°C. The maximum permeabilization was given by the fluorescence of the positive control.

### Cytotoxicity assays.

Human airway epithelial NCI-H292 cells (ATCC, Manassas, VA, USA) were cultured in RPMI 1640 medium supplemented with 200 mM l-glutamine, 10% (vol/vol) fetal calf serum, 100 IU/ml penicillin, 100 μg/ml streptomycin, and 2.5 mg/liter glucose and buffered with 25 mM HEPES at 37°C in a humidified, 5% CO_2_ water-jacketed incubator. Then, the cells were cultured overnight under serum-free conditions and treated with the peptides (5 or 20 μM). After 24 h, the cell viability was assessed by measuring the release of lactate dehydrogenase (LDH) as previously reported (22).

### Assays of cytokine production.

Alveolar macrophage cell line J774 cells (ATCC TIB-67, ATCC, Manassas, VA, USA) were plated in complete RPMI medium supplemented with 1% sodium pyruvate, 200 mM l-glutamine, 10% (vol/vol) fetal calf serum, 100 IU/ml penicillin, 100 μg/ml streptomycin, and 2.5 mg/liter glucose and buffered with 25 mM HEPES (pH 7.4). After 2 h, cells were incubated overnight with fresh medium and treated with peptides (5 or 20 μM) for 24 h. As a positive control for cytokine secretion, cells were infected with P. aeruginosa PAO1. After 1 h, bacteria were removed, the cells were reincubated in fresh culture medium for 20 h, and cytokine levels were measured by DuoSet enzyme-linked immunosorbent assay (ELISA) kits as previously described (24).

### Monitoring of peptide solubility.

Stationary-phase cultures of S. aureus SG511 grown at 37°C in LB medium were washed twice and resuspended in KB supplemented with 150 mM NaCl. Bacteria (10^8^ CFU/ml) were treated with 5 μM *Cg*-BigDef1[1–93], *Cg*-BigDef1[1–42], or *Cg*-BigDef1[44–93]. For negative controls, we used a same volume of water or 10% dimethyl sulfoxide (DMSO). After incubation for 30 min at 20°C, bacteria were removed by centrifugation and TFA-acidified supernatants were analyzed by HPLC on an Agilent HPLC HP-1290 system (as described above), with an Accucore C_4_ column (2.1 mm by 150 mm; flow rate, 300 μl/min); solvent A was 0.05% TFA–water, and solvent B was 0.04% TFA–MeCN. The gradient consisted of an increase from 2% solvent B to 60% in 30 min, and UV detection was performed at 225 nm.

### Immunofluorescence.

Overnight cultures of S. aureus SG511 were washed three times to remove all traces of culture media and adjusted to 10^7^ CFU/ml in killing buffer (KB) before contact with *Cg*-BigDef1[1–93] (5 μM final concentration). After 30 min at 25°C, bacterial cells were washed three times in KB, centrifuged onto glass slides (10 min, 1,500 rpm), and fixed for 10 min in PBS containing 4% paraformaldehyde. After permeabilization (0.01% Triton X-100, 10 min), cells were immunostained with a polyclonal mouse anti-*Cg-*BigDef1 antibody generated against the synthetic form of *Cg*-BigDef1[1–93]. Cells were incubated successively for 2 h with blocking solution, a 1:500 dilution of anti-*Cg-*BigDef1 antibody (or preimmune serum), and a 1:1,000 dilution of secondary anti-mouse antibody coupled to Alexa Fluor 488 (Invitrogen). After three washes in PBS containing 0.05% Tween 20, cells were stained for 10 min with 0.25 μg/ml DAPI (4′,6-diamidino-2-phenylindole). Coverslips were mounted with fluorescent mounting medium (Dako). Slides were observed with 63× objectives, and images were captured using a Leica TCS SPE confocal scanning laser microscope.

### Scanning electron microscopy of nanonets.

Overnight cultures of S. aureus SG511 or P. aeruginosa ATCC 9027 were washed three times in killing buffer supplemented with 150 or 300 mM NaCl. Bacterial suspensions adjusted to an *A*_600_ of 3 were deposited on a glass slide onto peptides (5 μM final concentration). After 24 h of incubation in a humid chamber, preparations were fixed with 2.5% glutaraldehyde. Fixed samples were dehydrated using a graded ethanol series (30% to 100%), followed by 10 min in graded ethanol-hexamethyldisilazane and then hexamethyldisilazane alone. Subsequently, the samples were sputter coated with an approximately 10-nm-thick gold film and then examined under a scanning electron microscope (Hitachi S4000; microscopy performed at CoMET, MRI-RIO Imaging, Biocampus, INM Montpellier, Montpellier, France) using a lens detector with an acceleration voltage of 10 kV at calibrated magnifications.

### Data availability.

Assignments were deposited as Biological Magnetic Resonance Bank (BRMB) entries 34345 and 34346 for *Cg*-BigDef1[44–93] and *Cg*-BigDef1[1–93], respectively. Coordinates were deposited as PDB entries 6QBK and 6QBL for *Cg*-BigDef1[44–93] and *Cg*-BigDef1[1–93], respectively.
