# Supplementary material for: The Ancestral N-Terminal Domain of Big Defensins Drives Bacterially Triggered Assembly into Antimicrobial Nanonets
Source: mBio. 2019 Oct 22;10(5):e01821-19. doi: 10.1128/mBio.01821-19 (PMC6805989; doi:10.1128/mBio.01821-19)
Supplement: FIG S1 [file mBio.01821-19-sf001.docx]

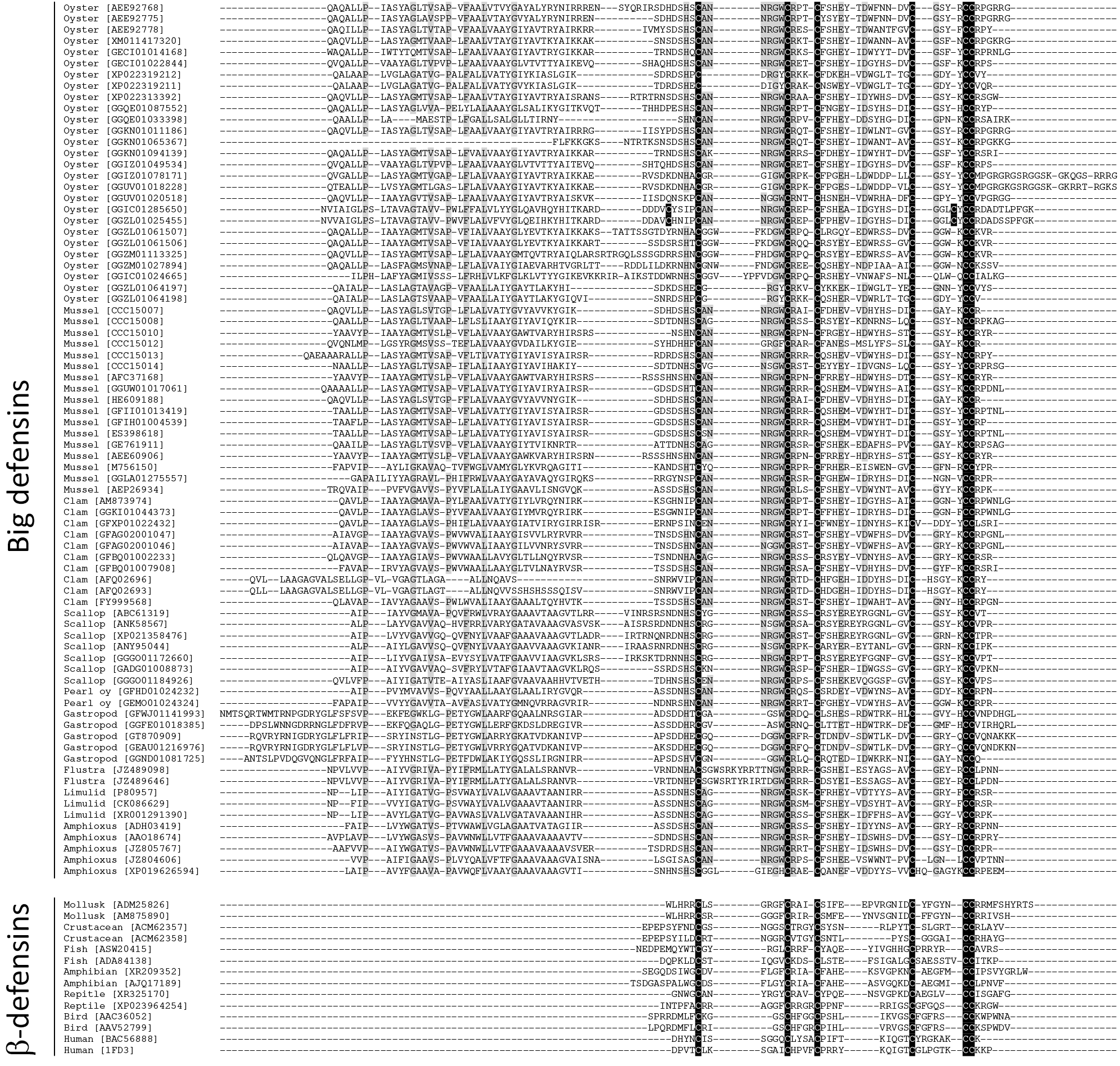


**Fig. S1**. Multiple amino acid sequence alignments of big defensins (Lophotrochozoa, Arthropoda and Cephalochordata) with β-defensins from both vertebrates (from fish to mammals) and invertebrates (mollusks and crustaceans). Conserved residues and cysteines are highlighted in grey and black, respectively
