# Supplementary material for: The Ancestral N-Terminal Domain of Big Defensins Drives Bacterially Triggered Assembly into Antimicrobial Nanonets
Source: mBio. 2019 Oct 22;10(5):e01821-19. doi: 10.1128/mBio.01821-19 (PMC6805989; doi:10.1128/mBio.01821-19)
Supplement: FIG S2 [file mBio.01821-19-sf002.docx]

**Peptide synthesis**

1-General information

All reagents and solvents were used without further purification. Protected amino acids, Rink’s linker and HCTU were purchased from Merck Biosciences (Nottingham, UK). Aminomethyl TentaGel R resin was purchased from Rapp polymers (Tübingen, Germany). Peptide synthesis grade DMF was obtained from VWR (Fontenay-sous-Bois, France). Ultrapure water was obtained using a Milli-Q water system from Millipore (Molsheim, France). All other chemicals were from Sigma Aldrich (St-Quentin-Fallavier, France) and solvents from SDS-Carlo Erba (Val de Reuil, France).

HPLC analyses and semi-preparative purifications were carried out on a LaChrom Elite system equipped with a Hitachi L-2130 pump, a Hitachi L-2455 diode array detector and a Hitachi L-2200 autosampler. Either Jupiter C4 (300 Å, 5 μm, 250 × 4.6 mm, 1 mL/min flow rate) or Chromolith High Resolution RP-18e (150 Å, 10 × 4.6 mm, 3 mL/min flow rate) columns were used for analysis and Jupiter C4 (300 Å, 5 μm, 250 × 10 mm, 3 mL/min flow rate, 70°C) ) or Nucleosil C18 (300 Å, 5 μm, 250 × 10 mm, 3 mL/min flow rate) for purification. Solvents A and B are 0.1 % TFA in H_2_O and 0.1 % TFA in MeCN, respectively.

LC-HRMS analyses were carried out on a Ultimate® 3000 RSLC HPLC system (Dionex, Germering, Germany), coupled with a QTOF mass the maXis mass spectrometer (maXisTM Bruker,) and fitted with a Zorbax 300 SB-C18 RRHD (300 Å, 1.8 μm, 100 × 2.1 mm, 0.5 mL/min flow rate, 40°C) column. Solvents A and B were 0.1 % formic acid in H_2_O and 0.08 % formic acid in MeCN, respectively. Gradient: 3 % B/A for 0.6 min, then 3 to 50 % B/A over 10.8 min. . The multiply-charged envelope was deconvoluted using the Charge Deconvolution algorithm in Bruker Data Analysis 4.1 software to obtain the monoisotopic [M] value.

The purified peptides dissolved in 8:2:0.01 H2O/MeCN/TFA were quantified by UV spectrometry at 280 nm, based on the following epsilons: ε_Tyr_ = 1290 L. mol^-1^.cm^-1^; ε_Trp_ = 5500 L. mol^-1^.cm^-1^; ε_cystine_ = 125 L. mol^-1^.cm^-1^.

2- General procedures for solid phase peptide synthesis

Fmoc-based solid phase peptide syntheses (SPPS) were carried out on a Prelude synthesizer from Protein Technologies. Standard side-chain protecting groups were used: Arg(Pbf), Asn(Trt), Asp(O*t*Bu), Cys(Trt), Glu(O*t*Bu), Gln(Trt), His(Trt), Ser(*t*Bu), Thr(*t*Bu), Trp(Boc) and Tyr(*t*Bu). Syntheses were performed at a 25 µmol scale starting from aminomethyl TentaGel R. Protected amino acids (0.25 mmol, 10 equiv.) were coupled using HCTU (98 mg, 0.238 mmol, 9.5 equiv.) and *i*Pr_2_NEt (87 µL, 0.5 mmol, 20 equiv.) in NMP (3 mL) for 30 min. Capping of eventual unreacted amine groups was achieved by treatment with acetic anhydride (143 µL, 1.51 mmol, 60 equiv.), *i*Pr_2_NEt (68 µL, 0.39 mmol, 15.5 equiv.) and HOBt (6 mg, 0.044 mmol, 1.8 equiv.) in NMP (3 mL) for 7 min. Fmoc group was removed by three successive treatments with 20 % piperidine in NMP (3 mL) for 3 min.

The crude peptides were deprotected and cleaved from the resin through a treatment with TFA:H_2_O:*i*Pr_3_SiH:phenol, 88/5/2/5 for 3 h, then precipitated by dilution into an ice-cold 1:1 (v/v) diethyl ether/petroleum ether mixture, recovered by centrifugation and further washed three times with diethyl ether.

3- Syntheses of separate domains *Cg*-BigDef1[1-42] and *Cg*-BigDef1[44-93] S3

3-1 *Cg*-BigDef1[1-42]


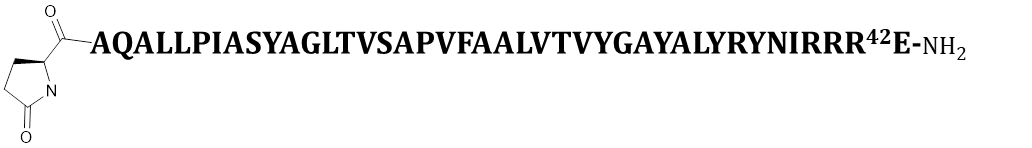


ε = 6450 M^-1^cm^-1^ in H_2_O/MeCN (8:2) containing 0.1% TFA

|  |  |
| --- | --- |
| **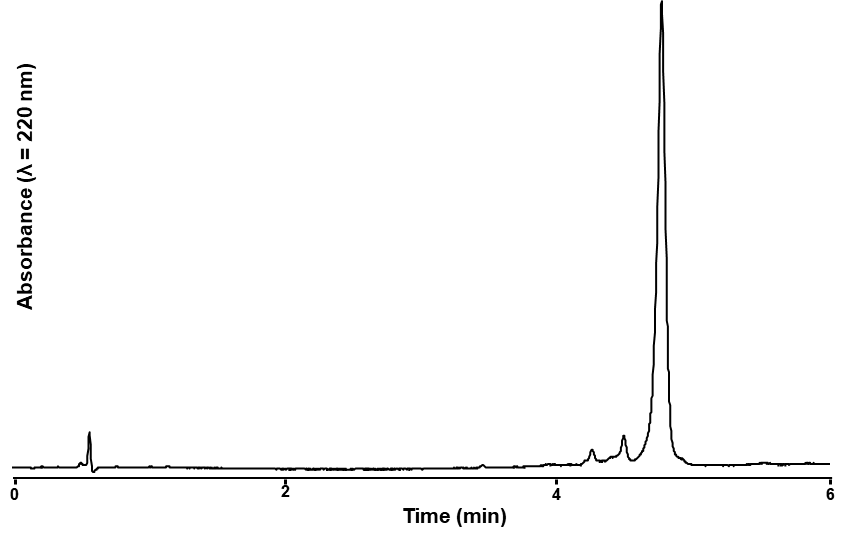**  **Fig S2a**: HPLC trace of purified *Cg*-BigDef1[1-42].  Chromolith RP-C18, 3 mL/min, A = 0.1% TFA in water, B = 0.1% TFA in CH_3_CN, gradient B in A : 5-50 over 5min.  ESI-HR-MS: found 4597.4953(calculated for C_214_H_333_N_57_O_56_ 4597.4962 Da, monoisotopic mass) |  |

3-1 *Cg*-BigDef1[44-93]


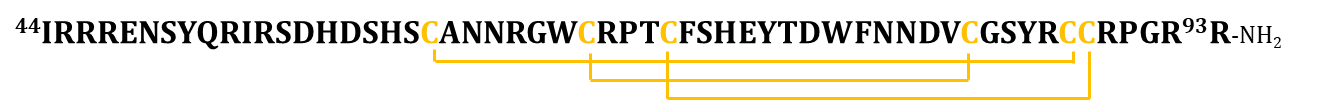


ε : 15245 M^-1^ cm^-1^ in H_2_O/MeCN (8:2) containing 0.1% TFA

Purified reduced form of *Cg*-BigDef1[44-93] before oxidative folding (blue trace, Fig. S2b)

ESI-HR-MS: 5996.6034 (calculated for C_249_H_366_N_88_O_76_S_6_ 5996.5804 Da, monoisotopic mass)

The reduced form of *Cg*-BigDef1[44-93] (blue trace, Fig S2b) was purified by HPLC and then engaged in oxidative folding. The oxidative folding was performed at a peptide/GSH/GSSG molar ratio of 1:100:10 in a deoxygenated MeCN/200 mM Tris-HCl buffer, pH 8.5 solution (25:75, v/v), containing 1 mM EDTA, and under argon atmosphere. The peptide concentration (20 µM/ml) was measured using UV spectrophotometry at 280 nm. The kinetics of the oxidative folding was monitored by analytical C18 reversed phase HPLC (Fig. S2b). Aliquots (100 µl) of the folding reaction mixture were taken at regular intervals, and the reaction was stopped by adding 2 µl of neat TFA before HPLC analysis. The oxidative folding was nearly quantitative over 16 h (pink trace, Fig. 2b). After acidification of the reaction mixture with neat TFA down to pH 4, the reaction mixture was concentrated under vacuum and the target peptide was purified on to a C18 column and lyophilized (Fig. S2c).


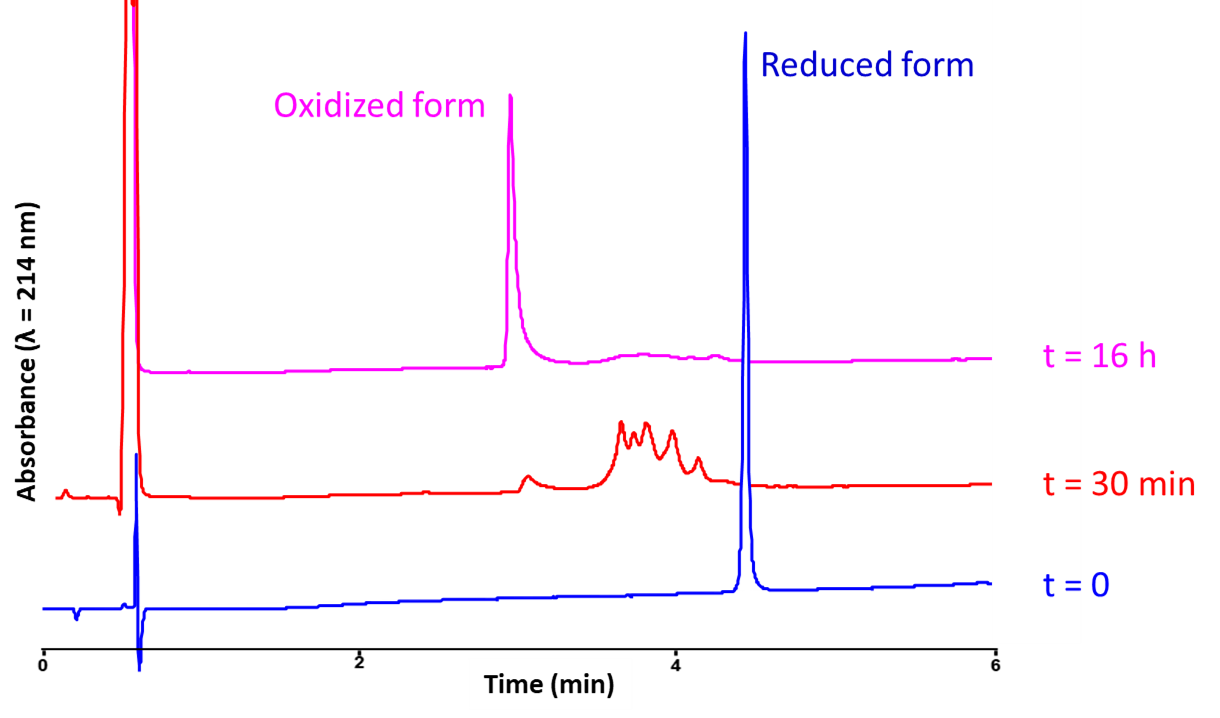


**Fig. S2b**: Analytical monitoring of the *Cg*-BigDef1[44-93] oxidative folding by HPLC.

Chromolith RP C18, 3 mL/min, A = 0.1% TFA in water, B = 0.1% TFA in CH_3_CN, gradient B in A : 15-40 over 5min.


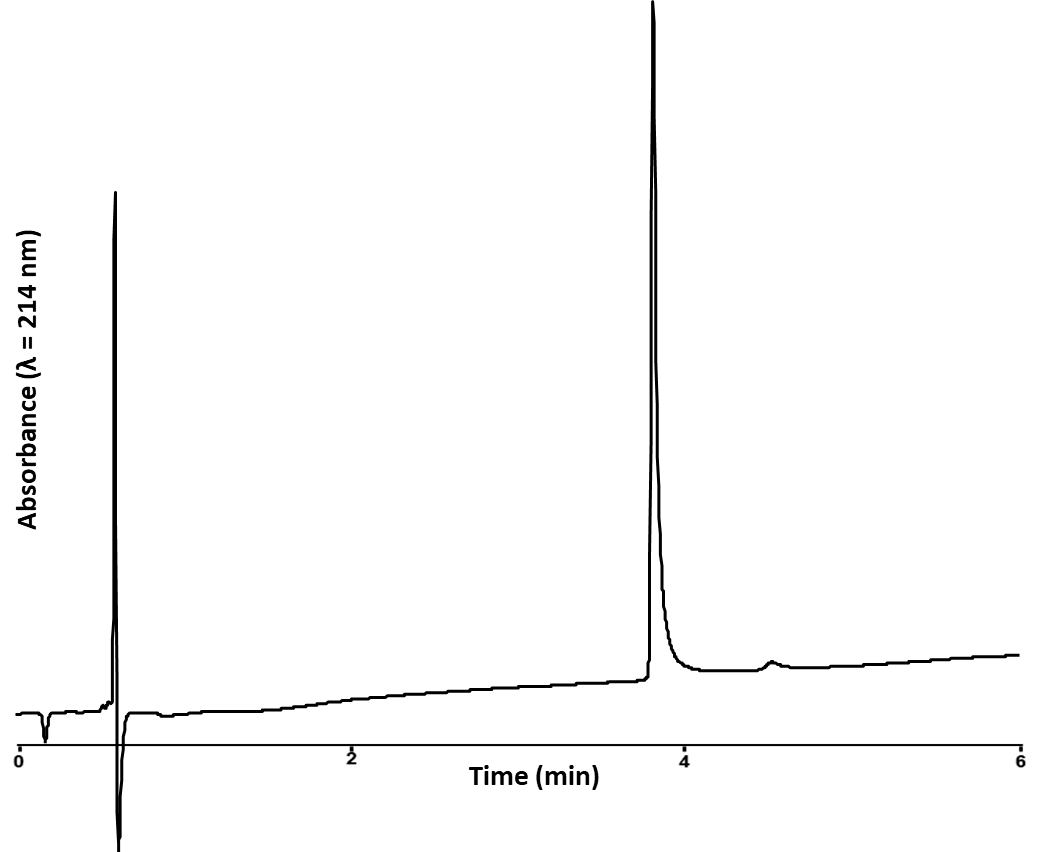


**Fig S2c**: HPLC trace of purified *Cg*-BigDef1[44-93].

Chromolith RP-C18, 3 mL/min, A = 0.1% TFA in water, B = 0.1% TFA in CH_3_CN, gradient B in A : 5-50 over 5min.

Purified oxidized form of *Cg*-BigDef1[44-93] (Fig. S2c)

ESI-HR-MS: found 5990.5315 (calculated for C_249_H_360_N_88_O_76_S_6_ 5990.5336Da, monoisotopic mass)

4- Synthesis of *Cg*-BigDef1[1-93]


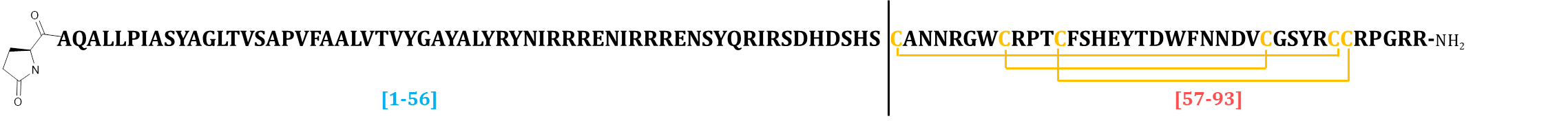


4-1 Synthesis of the reduced form by SPPS then NCL


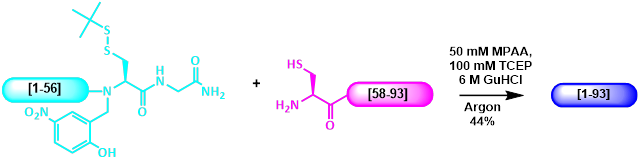


The synthesis of the reduced form of *Cg*-BigDef1[1-93] by native chemical ligation (NCL) using our *N*-Hnb-Cys (2-hydroxy-4-nitro-benzyl-cysteine) thioesterification device has already been reported (see Terrier *et al*, Chem. Sci., 2016, 7, 339-345 for details) and was scaled up in this work.

Under argon, 2.65 mL of a degassed 0.4 M pH 6.3 sodium phosphate buffer containing 50 mM MPAA, 100 mM TCEP and 6 M guanidine hydrochloride were added to 40.35 mg of the *Cg*-BigDef1 [1-56]-(Hnb)C(S*t*Bu)G-NH_2_ (5.29 μmol, final concentration 2 mM) and 30 mg of the *Cg*-BigDef1 [57-93] (5.7 μmol, 1.8 equiv., final concentration 2.16 mM). The ligation was carried out at 37°C under slight horizontal shaking and monitored by RP-HPLC (Chromolith, gradient: 20-70 % B/A over 6 min. After 48 h, the reaction mixture was acidified with a 90 :10 6 M guanidine hydrochloride/AcOH (v/v) mixture (1.32 mL) followed by 4 mL of 65:35 H_2_0/MeCN (v/v) containing 0.1% TFA in water. Under these conditions, no precipitation was observed. The ligation product (the reduced form of *Cg*-BigDef1[1-93]) was purified by semi-preparative RP-HPLC (Nucleosil, gradient: 50-60 % B/A over 10 min). 2.34 µmol were obtained (yield = 44 %, UV titration at 280 nm, molar extinction coefficient = 21320 L.mol-1.cm−1).

4-2 Synthesis of the oxidized form of the *Cg*-BigDef1[1-93]

The HPLC-purified reduced form of *Cg*-BigDef1[1-93] (2.34 µmol, 10 µM final) (blue trace, Fig S2d) was engaged in oxidative folding under argon atmosphere. The oxidative folding was performed at a peptide/GSH/ GSSG molar ratio of 1:100:10 in deoxygenated MeCN/200 mM Tris-HCl buffer, pH 8.5 solution (50:50, v/v), containing 1 mM EDTA, and under an argon atmosphere. The kinetics of the oxidative folding was monitored by analytic C18 reversed phase HPLC (Fig. S2b). Aliquots (100 µl) of the folding reaction mixture were taken at regular intervals, and the reaction was stopped by adding 2 µl of neat TFA before HPLC analysis. The oxidative folding was nearly quantitative over 48 h (green trace, Fig. 2d). Note that the oxidized form is rather unusually eluted later than the reduced one, indicating a hydrophobic feature possibly related to the hydrophobic nature of the 3D fold revealed by the NMR analysis (see main text). After acidification of the reaction mixture with neat TFA up to pH 4, the reaction mixture was concentrated under vacuum and the target peptide was purified on a Jupiter C4 column at 70 °C and lyophilized (1.09 µmol, isolated yield 46%) (Fig. S2e).

**
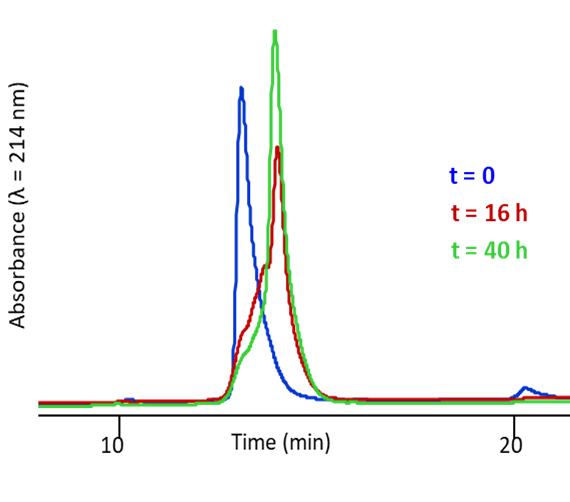
**

*m/z*

B

A

**Fig S2d**: Analytical monitoring of *Cg*-BigDef1[1-93] oxidative folding by HPLC.

A: Jupiter RP-C4, 1 mL/min, A = 0.1% TFA in water, B = 0.1% TFA in CH_3_CN, gradient B in A : 30-80 over 30 min at 70 °C. Blue trace: HPLC-purified reduced form of Cg-BigDef1[1-93] before oxidative folding, red trace after 16h reaction, green trace after 40h reaction.

B: ESI-HR-MS of the reduced form of *Cg*-BigDef1[1-93]: found found 10691.0918 (calculated for C_467_ H_702_ N_146_ O_134_ S_6_ 10691.0930 Da, monoisotopic mass).

B

A

**
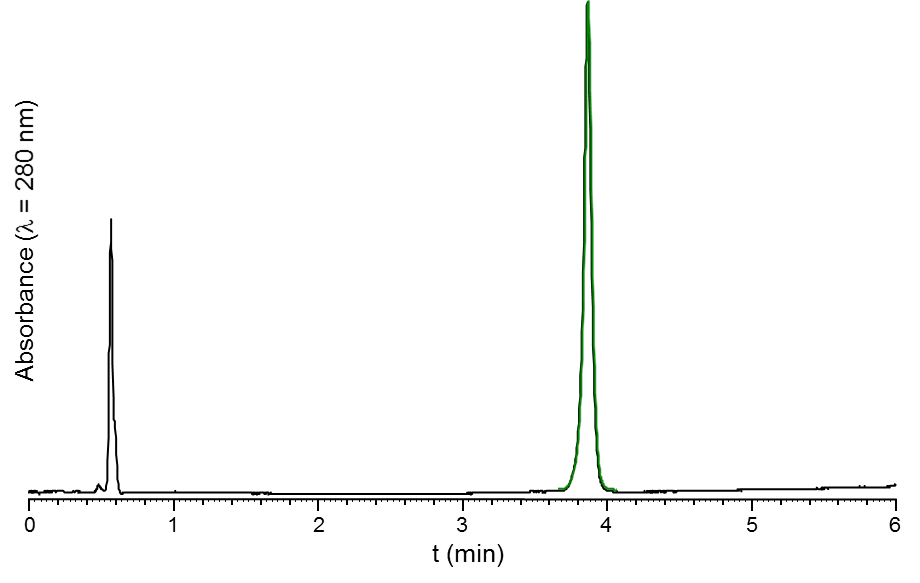
**


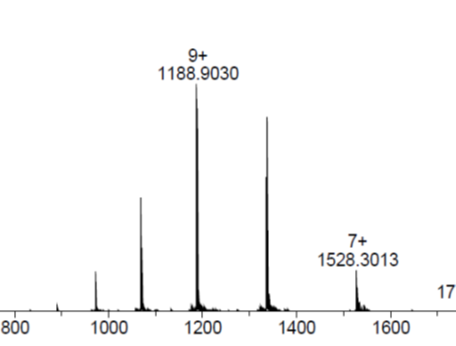


*m/z*

Time (min)

**Fig S2e**: HPLC and MS characterization of purified oxidized form of *Cg*-BigDef1[1-93].

A/ HPLC trace of purified *Cg*-BigDef1[1-93] (Chromolith RP-C18, 3 mL/min, A = 0.1% TFA in water, B = 0.1% TFA in CH_3_CN, gradient B in A : 30-80 over 6 min).

B/ ESI-HR-MS of the oxidized form of *Cg*-BigDef1[1-93]: found 10685.0402 (calculated for C_467_ H_696_ N_146_ O_134_ S_6_ 10685.0460 Da , monoisotopic mass).
