# Supplementary material for: The Ancestral N-Terminal Domain of Big Defensins Drives Bacterially Triggered Assembly into Antimicrobial Nanonets
Source: mBio. 2019 Oct 22;10(5):e01821-19. doi: 10.1128/mBio.01821-19 (PMC6805989; doi:10.1128/mBio.01821-19)
Supplement: FIG S3 [file mBio.01821-19-sf003.docx]

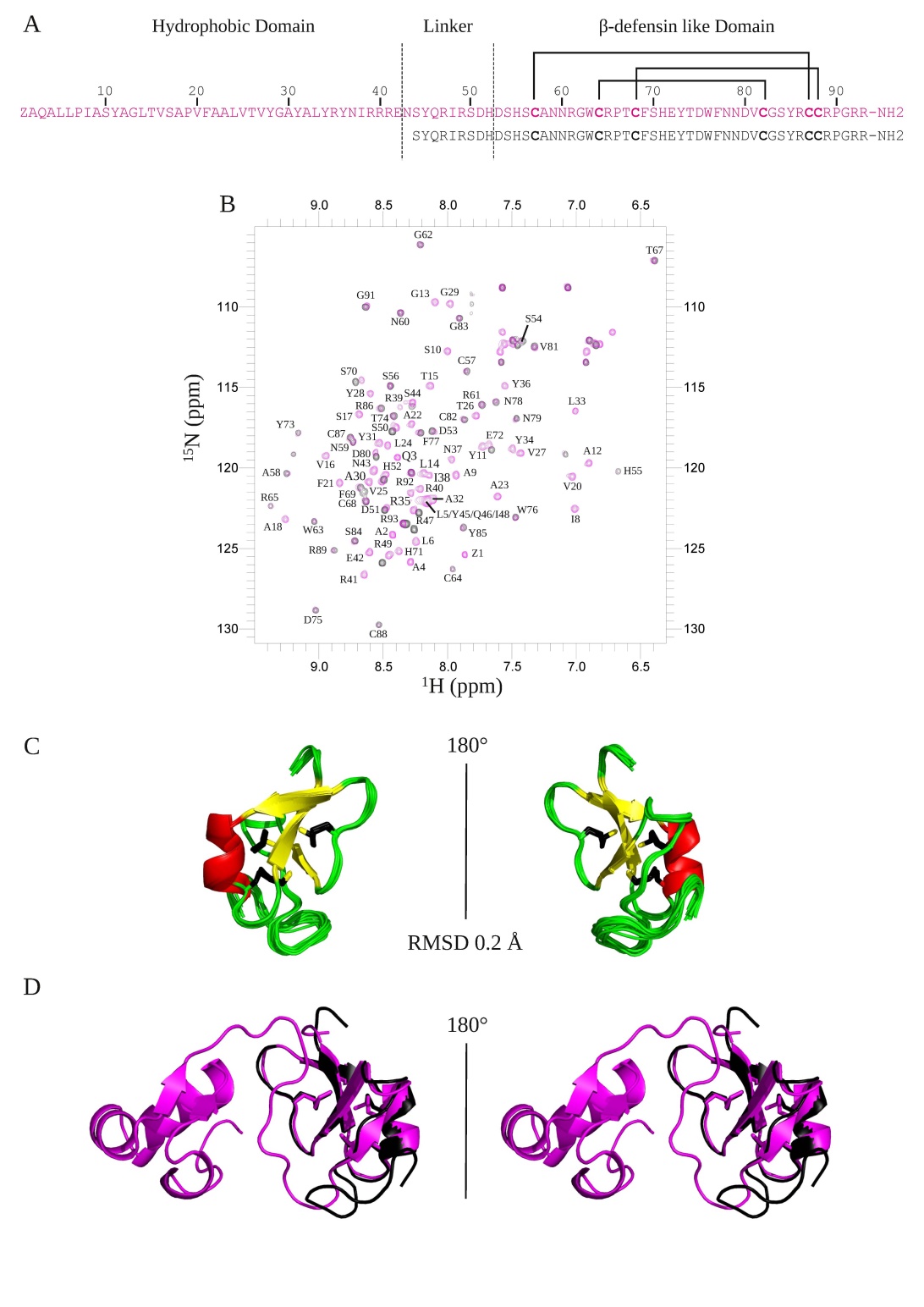


**Fig. S3. *Cg*-BigDef1[44-93] global fold and comparison with the one of *Cg*-BigDef1[1-93].**

A. *Cg*-BigDef1[44-93] (black) and *Cg*-BigDef1[1-93] (magenta) primary structures. Cysteine residues are indicated in bold and their pairings are indicated by black lines. B. Overlay of ^15^N- sofast-HMQC of *Cg*-BigDef1[44-93] (black) and *Cg*-BigDef1[1-93] (magenta). The assignment of *Cg*-BigDef1[1-93] is reported. Both peptides were highly structured as shown by a good dispersion of the amide chemical shifts in their ^1^H NMR and sofast-HMQC spectra. C. Superimposition of the 10 models representative of *Cg*-BigDef1[44-93] solution structure. D. Structure alignment of *Cg*-BigDef1[44-93] (black) and *Cg*-BigDef1[1-93] (magenta).
