# Supplementary material for: The Ancestral N-Terminal Domain of Big Defensins Drives Bacterially Triggered Assembly into Antimicrobial Nanonets
Source: mBio. 2019 Oct 22;10(5):e01821-19. doi: 10.1128/mBio.01821-19 (PMC6805989; doi:10.1128/mBio.01821-19)
Supplement: TABLE S2 [file mBio.01821-19-st002.docx]

**Table S2. NMR constraints and structural statistics for *Cg-*BigDef1[44-93].**

| **NMR constraints** | |
| --- | --- |
| **Distance restraints** | |
| Total NOE | 1568 |
| Unambiguous | 1376 |
| Ambiguous | 192 |
| Hydrogen bonds | 13 |
| **Disulfide bridges^a^** | 3 |
|  |  |
| **Structural Statistics (6QBK.pdb)** | |
| **Average violations per structure** | |
| NOEs ≥ 0.3 Å | 0 |
| Hydrogen bonds ≥ 0.5 Å | 0 |
| Average pairwise rmsd (Å) | 0.2 |
| **Ramachandran Analysis** | |
| Most favored region and allowed region | 95.1% |
| Generously allowed | 1.2% |
| Disallowed | 3.7% |
| **Energies (kcal.mol ^-1^)^b^** | |
| Electrostatic | -1729 ± 37 |
| Van der Walls | -374 ± 8 |
| Total energy | -1562 ± 33 |
| Residual NOE energy | 28 ± 6 |

a: Introduced as ambiguous; b: values are given as mean ± standard deviation (n=10)
