# Supplementary material for: The Ancestral N-Terminal Domain of Big Defensins Drives Bacterially Triggered Assembly into Antimicrobial Nanonets
Source: mBio. 2019 Oct 22;10(5):e01821-19. doi: 10.1128/mBio.01821-19 (PMC6805989; doi:10.1128/mBio.01821-19)
Supplement: TABLE S3 [file mBio.01821-19-st003.docx]

**Table S3. Strains and media**

| **Strain** | **Status** | **Comment** | **Culture medium for antimicrobial assays** | **Origin** | **Reference** |  |
| --- | --- | --- | --- | --- | --- | --- |
| **Gram-negative bacteria** |  |  |  |  |  |  |
| *Aliivibrio fischerii*  7P_21 | Env | Isolated from the water column (Atlantic coast, bay of the city of Brest, France). | Zobell 1/3 | F. Le Roux (Roscoff, France) | Bruto et al. 2016 |  |
| *Burkholderia multivorans*  12/11/13-B-2333 | Clin/h | Isolated from a 23-year-old Cystic fibrosis patient. Multidrug resistant (resistance to all antibiotics tested). | Poor broth | Cystic fibrosis center, Montpellier Hospital, France |  |  |
| *Escherichia coli*  MC4100, ATCC 35695 | Ref |  | Poor broth | ATCC |  |  |
| *Pseudomonas aeruginosa*  ATCC 9027 | Ref | Outer ear infection | Poor broth | ATCC |  |  |
| *Pseudomonas aeruginosa*  (Pa02) 12/07/11-B-2285 | Clin/h | Isolated from a 49-year-old Cystic fibrosis patient. Mucoid strain. Low level of non enzymatic acquired resistance. | Poor broth | Cystic fibrosis center, Montpellier Hospital, France | Michon et al. 2014 |  |
| *Pseudomonas aeruginosa*  (Pa25) 13/07/11-B-3003 | Clin/h | Isolated from 17-year-old Cystic fibrosis patient. Non-mucoid strain. Wild-type antimicrobial resistance phenotype. | Poor broth | Cystic fibrosis center, Montpellier Hospital, France | Michon et al. 2014 |  |
| *Vibrio breoganii*  7F1_16 | Env | Isolated from the water column (Atlantic coast, bay of the city of Brest, France). | Zobell 1/3 | F. Le Roux (Roscoff, France) | Bruto et al. 2016 |  |
| *Vibrio crassostreae*  7F5_29 | Clin/o | Isolated from diseased oysters (Atlantic coast, bay of the city of Brest, France). | Zobell 1/3 | F. Le Roux (Roscoff, France) | Bruto et al. 2016 |  |
| *Vibrio crassostreae*  7T4_12 | Clin/o | Isolated from diseased oysters (Atlantic coast, bay of the city of Brest, France). | Zobell 1/3 | F. Le Roux (Roscoff, France) | Bruto et al. 2016 |  |
| *Vibrio harveyi*  7G5_1 | Clin/o | Isolated from diseased oysters (Atlantic coast, bay of the city of Brest, France). | Zobell 1/3 | F. Le Roux (Roscoff, France) | Bruto et al. 2016 |  |
| *Vibrio orientalis*  8F5_42 | Env | Isolated from the water column (Atlantic coast, bay of the city of Brest, France). | Zobell 1/3 | F. Le Roux (Roscoff, France) | Bruto et al. 2016 |  |
| *Vibrio tasmaniensis*  3T8_11 | Clin/o | Isolated from diseased oysters (Atlantic coast, bay of the city of Brest, France). | Zobell 1/3 | F. Le Roux (Roscoff, France) | Bruto et al. 2016 |  |
| *Vibrio tasmaniensis*  7G7_3 | Clin/o | Isolated from diseased oysters (Atlantic coast, bay of the city of Brest, France). | Zobell 1/3 | F. Le Roux (Roscoff, France) | Bruto et al. 2016 |  |
| *Vibrio tasmaniensis*  LGP32 | Clin/o | Isolated from diseased oysters (Atlantic coast, bay of the city of Brest, France). | Zobell 1/3 | F. Le Roux (Roscoff, France) | Gay et al. 2004 |  |
| **Gram-positive bacteria** |  |  |  |  |  |  |
| *Corynebacterium stationis*  CIP 101282 | Ref | Film of marine fouling organisms | Zobell 1/3 | Pasteur Institute (Pasteur, France) |  |  |
| *Microbacterium maritypicum*  CIP 105733^T^ | Ref | Seawater and marine mud | Zobell 1/3 | Pasteur Institute (Pasteur, France) |  |  |
| *Micrococcus luteus*  CIP 53.45 | Ref | Renamed *Kocuria rhizophila* | Poor broth | Pasteur Institute (Pasteur, France) |  |  |
| *Staphylococcus aureus*  Newman | Ref | Human infection | Poor broth |  |  |  |
| *Staphylococcus aureus*  SG511 | Ref |  | Poor broth | H.G. Sahl (Bonn, Germany) |  |  |
| *Staphylococcus aureus*  (MSSA)  07/02/14-B-5264 | Clin/h | Isolated from a 12-year-old Cystic fibrosis patient. Methicillin-susceptible, resistant to penicillin G and amoxicillin through penicillinase production, resistant to erythromycin. | Poor broth | Montpellier Hospital, France |  |  |
| *Staphylococcus aureus*  (MRSA)  31/01/14*-*B-5284 | Clin/h | Isolated from a 4-year-old Cystic fibrosis patient. Multidrug resistance: methicillin-resistant, resistant to kanamycin, tobramycin, erythromycin, lincomycin, ofloxacin. | Poor broth | Cystic fibrosis center, Montpellier Hospital, France |  |  |
| *Staphylococcus aureus*  (MRSA, GISA)  24/11/08-B-1347 | Clin/h | Isolated from a 21-year-old patient hospitalized in intensive care unit. Multidrug resistance: methicillin-resistant, resistant to kanamycin, tobramycin, gentamicin, tetracyclin, erythromycin, lincomycin, ofloxacin, rifampin, fosfomycin and Intermediate susceptibility to glycopeptides. | Poor broth | Montpellier University Hospital, France |  |  |
| *Staphylococcus aureus*  #53863  (MRSA) | Clin/h | Isolated from a 24-year-old cystic fibrosis patient. Methicillin-resistant. | Poor broth | Dr. P.C. Morand (Cochin Hospital, Paris, France) |  |  |
| *Staphylococcus aureus*  (MRSA)  #7877 | Clin/h | Isolated from a 54-year-old cystic fibrosis patient. Multidrug resistance: methicillin-resistant, resistant to erythromycin.. | Poor broth | Dr. P.C. Morand (Cochin Hospital, Paris, France) |  |  |
| Env: environmental isolate; Clin: clinical isolate, either from human (h) or diseased oyster (o) origin; Ref: reference strain. Comment on and origin of reference strains has been indicated where available.  CIP: Collection de l’Institut Pasteur; ATCC: American Type Culture Collection; MSSA: Methicillin-susceptible *Staphylococcus aureus*; MRSA: Methicillin-resistant *Staphylococcus aureus*; GISA: Glycopeptide-Intermediate *Staphylococcus aureus.* Zobell 1/3 medium (400mM NaCl, 20mM KCl, 5mM MgSO4, 1.4mM CaCl2, 1.3g/L bactopeptone and 0.33g/L yeast extract). Poor broth medium (1% bactotryptone, 0.5% NaCl w/v, pH 7.5). | | | | | | |
