# Supplementary material for: The Ancestral N-Terminal Domain of Big Defensins Drives Bacterially Triggered Assembly into Antimicrobial Nanonets
Source: mBio. 2019 Oct 22;10(5):e01821-19. doi: 10.1128/mBio.01821-19 (PMC6805989; doi:10.1128/mBio.01821-19)
Supplement: FIG S4 [file mBio.01821-19-sf004.docx]

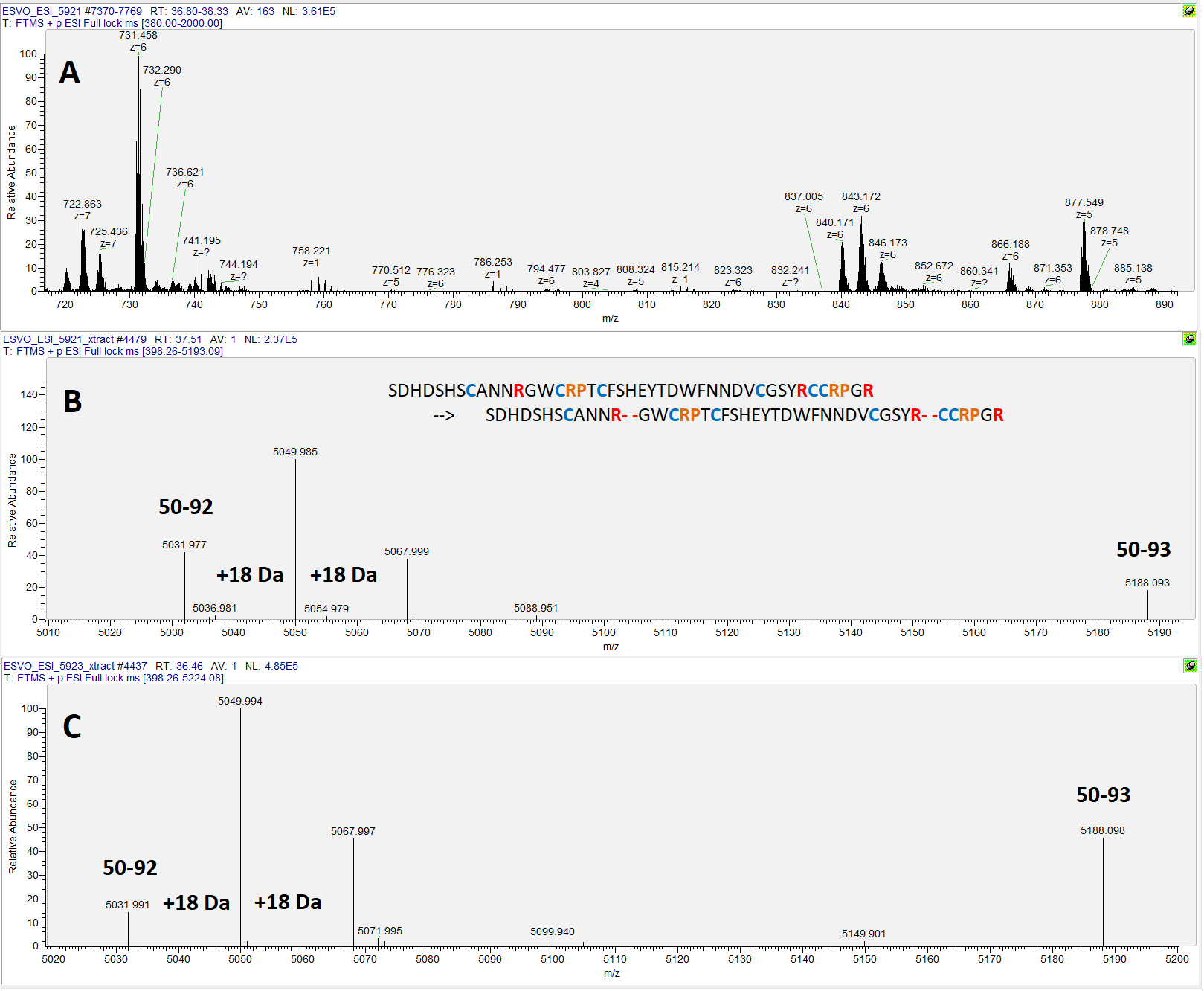


**Fig. S4: Accurate mass determination of the 50-92 tryptic fragments from *Cg*-BigDef1 [1-93] and *Cg*-BigDef1 [44-93]**

The tryptic digest of *Cg*-BigDef1 [1-93], obtained in non-reducing conditions in the presence of 1% RapiGest surfactant, was analyzed by LC-ESI-MS, using a high-resolution Q-Exactive Orbitrap, and the resulting datafile was processed in order to translate the observed multicharged peptide ions (panel A) into the corresponding monoisotopic mass of the compounds at the origin of these ions (panel B). Based on their mass, two observed values are likely corresponding to the tryptic peptides 50-93 (observed mass 5,188.093 Da) and the tryptic fragment 50-92 (observed mass 5,031.977 Da), with all six cysteine residues involved in disulfide bonds. Additionally, two compounds were accompanying the peptide ions of the fragment 50-92, and were resolved into the masses 5,049.985 Da and 5,067.999 Da, showing each time an addition of 18 Da. These two ions are likely corresponding to the result of the hydrolysis by trypsin of either one or two amide bonds after the available arginine in this part of the protein sequence, as shown in the text inserted in panel B.  The same process applied to *Cg*-BigDef1 [44-93] resulted in a similar spectrum (panel C).
