# Supplementary material for: The Ancestral N-Terminal Domain of Big Defensins Drives Bacterially Triggered Assembly into Antimicrobial Nanonets
Source: mBio. 2019 Oct 22;10(5):e01821-19. doi: 10.1128/mBio.01821-19 (PMC6805989; doi:10.1128/mBio.01821-19)
Supplement: FIG S5 [file mBio.01821-19-sf005.docx]

**
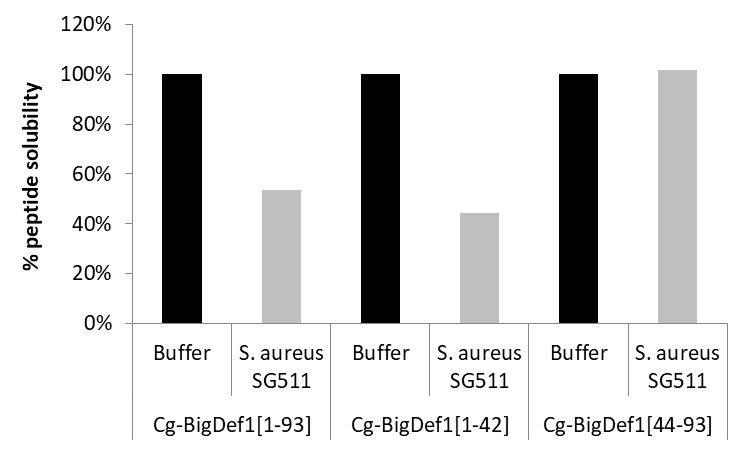
**

**Fig. S5 : Loss of *Cg-*BigDef1[1-93] and *Cg-*BigDef1[1-42] solubility after 30min of contact with *S. aureus.***

*Cg-*BigDef1[1-93], *Cg-*BigDef1[1-42] or *Cg-*BigDef1[43-93] were added (5µM final concentration) to 200µL of a solution of Tris-HCl 100mM pH 8, CaCl_2_ 1mM, NaCl 150mM containing a suspension of bacteria (optical density of 0.1). After 30min at 20°C, bacteria were pelleted by centrifugation and the supernatant collected. In control tubes, peptides were added to 200µL of buffer only. After acidification by TFA, 90µL of supernatant were injected on HPLC and the peak area (OD225nm) for the present *Cg-*BigDef1 peptide were measured. The above graphs show the peak areas from bacteria samples in proportion of the area obtained for controls (buffer+peptides). It is considered that peptides have retained their entire solubility in buffer.
