# Supplementary material for: The Ancestral N-Terminal Domain of Big Defensins Drives Bacterially Triggered Assembly into Antimicrobial Nanonets
Source: mBio. 2019 Oct 22;10(5):e01821-19. doi: 10.1128/mBio.01821-19 (PMC6805989; doi:10.1128/mBio.01821-19)
Supplement: TEXT S1 [file mBio.01821-19-s0001.docx]

**NMR and Structure Calculations**

*Cg-*BigDef1[44-93] and *Cg-*BigDef1[1-93] were dissolved in H_2_O:D_2_O (9:1 ratio, v/v) at a concentration of 1.5 mM and 1.0 mM respectively. pH was adjusted to 4.6 for both samples. 2D ^1^H NOESY, 2D ^1^H TOCSY, a sofast-HMQC ([1](#_ENREF_1)) (^15^N natural abundance) and a ^13^C-HSQC (^13^C natural abundance) were performed at 298 K on an Avance III HD BRUKER 700 MHz spectrometer equipped with a cryoprobe. ^1^H chemical shifts were referenced to the water signal (4.77 ppm at 298 K). NMR data were processed using Bruker's Topspin 3.2^TM^ and analyzed with CCPNMR (version 2.2.2) ([2](#_ENREF_2)).

A complete assignment of ^1^H chemical shifts was obtained for both proteins by the analysis of 2D-TOCSY and NOESY spectra. Natural-abundance heteronuclear NMR spectra helped us to unambiguously assign ^1^H chemical shifts (BRMB entry 34345 and 34346 for *Cg-*BigDef1[44-93] and *Cg-*BigDef1[1-93], respectively).

Structures for both proteins were calculated using CNS ([3](#_ENREF_3), [4](#_ENREF_4)) through the automatic assignment software ARIA2 (version 2.3) ([5](#_ENREF_5)) with NOE derived distances, hydrogen bonds (in accordance with the observation of typical long or medium distance NOE cross peaks network for β-sheets and α-helices respectively – H^N^/H^N^, H^N^/H^α^, H^α^/H^α^) and 3 disulfide bridges (ambiguous distances restraints). For *Cg-*BigDef1[44-93], backbone dihedral angle restraints were added (determined with the DANGLE program ([6](#_ENREF_6))). The pyroglutamic acid residue (Z or Pca) located at the N-terminal position of *Cg-*BigDef1[1-93] sequence and the arginine carboxamide (AAR = R-NH_2_) located at the C-terminal position of *Cg-*BigDef1[44-93] and *Cg-*BigDef1[1-93] are considered as nonstandard residues in CNS. Topology libraries (topalldg5.3.pro and topalldg5.3.pep) were modified as described in the ARIA 2.3 tutorials.

The last run for *Cg-*BigDef1[1-93] was performed with 250 initial structures and 150 structures were refined in water. The 150 water-refined structures of *Cg-*BigDef1[1-93] possess three disulfide bridges with an identical pairing: Cys57-Cys87, Cys64-Cys82 and Cys68-Cys88, in agreement with the typical NOESY cross-peaks observed for disulfide bridges (H^α^/H^α^, H^α^/H^β^ and H^β^/H^β^) and with the pairing determined on the native *Tt*-BigDef ([7](#_ENREF_7), [8](#_ENREF_8)) . Among these 150 water-refined structures, ten were selected in agreement with all the experimental data and the standard covalent geometry. Restraints and coordinates were deposited as PDB entry 6QBL (see Table S1).

The last run for *Cg-*BigDef1[44-93] was performed with 500 initial structures and 200 structures were refined in water. Among them, 100 have an identical bonding pattern to the one of *Cg-*BigDef1[1-93]. Among them, 10 structures were selected, in agreement with all the experimental data, supported by the typical NOESY cross-peaks observed for disulfide bridges) and the standard covalent geometry. Restraints and coordinates were deposited as PDB entry 6QBK (see Table S2). The quality of final structures was evaluated using PROCHECK-NMR ([9](#_ENREF_9)) and PROMOTIF ([10](#_ENREF_10)). Electrostatic and hydrophobic potential were determined at the Connoly surface by using by APBS ([11](#_ENREF_11)) and the Platinum server ([12](#_ENREF_12)), respectively. The figures were prepared with PYMOL ([13](#_ENREF_13)).

**References**

1. Schanda P, Kupce E, Brutscher B. 2005. SOFAST-HMQC experiments for recording two-dimensional heteronuclear correlation spectra of proteins within a few seconds. J Biomol NMR 33:199-211.

2. Vranken WF, Boucher W, Stevens TJ, Fogh RH, Pajon A, Llinas M, Ulrich EL, Markley JL, Ionides J, Laue ED. 2005. The CCPN data model for NMR spectroscopy: development of a software pipeline. Proteins 59:687-96.

3. Brunger AT. 2007. Version 1.2 of the Crystallography and NMR system. Nat Protoc 2:2728-33.

4. Brunger AT, Adams PD, Clore GM, DeLano WL, Gros P, Grosse-Kunstleve RW, Jiang JS, Kuszewski J, Nilges M, Pannu NS, Read RJ, Rice LM, Simonson T, Warren GL. 1998. Crystallography & NMR system: A new software suite for macromolecular structure determination. Acta Crystallogr D Biol Crystallogr 54:905-21.

5. Rieping W, Habeck M, Bardiaux B, Bernard A, Malliavin TE, Nilges M. 2007. ARIA2: automated NOE assignment and data integration in NMR structure calculation. Bioinformatics 23:381-2.

6. Cheung MS, Maguire ML, Stevens TJ, Broadhurst RW. 2010. DANGLE: A Bayesian inferential method for predicting protein backbone dihedral angles and secondary structure. J Magn Reson 202:223-33.

7. Saito T, Kawabata S, Shigenaga T, Takayenoki Y, Cho J, Nakajima H, Hirata M, Iwanaga S. 1995. A novel big defensin identified in horseshoe crab hemocytes: isolation, amino acid sequence, and antibacterial activity. J Biochem 117:1131-7.

8. Tang YQ, Selsted ME. 1993. Characterization of the disulfide motif in BNBD-12, an antimicrobial beta-defensin peptide from bovine neutrophils. J Biol Chem 268:6649-53.

9. Laskowski RA, Rullmannn JA, MacArthur MW, Kaptein R, Thornton JM. 1996. AQUA and PROCHECK-NMR: programs for checking the quality of protein structures solved by NMR. J Biomol NMR 8:477-86.

10. Hutchinson EG, Thornton JM. 1996. PROMOTIF--a program to identify and analyze structural motifs in proteins. Protein Sci 5:212-20.

11. Baker NA, Sept D, Joseph S, Holst MJ, McCammon JA. 2001. Electrostatics of nanosystems: application to microtubules and the ribosome. Proc Natl Acad Sci U S A 98:10037-41.

12. Pyrkov TV, Chugunov AO, Krylov NA, Nolde DE, Efremov RG. 2009. PLATINUM: a web tool for analysis of hydrophobic/hydrophilic organization of biomolecular complexes. Bioinformatics 25:1201-2.

13. De Lano WL. 2002. Pymol, , South San Francisco, CA.
